# The chemiluminescence based Ziplex^® ^automated workstation focus array reproduces ovarian cancer Affymetrix GeneChip^® ^expression profiles

**DOI:** 10.1186/1479-5876-7-55

**Published:** 2009-07-06

**Authors:** Michael CJ Quinn, Daniel J Wilson, Fiona Young, Adam A Dempsey, Suzanna L Arcand, Ashley H Birch, Paulina M Wojnarowicz, Diane Provencher, Anne-Marie Mes-Masson, David Englert, Patricia N Tonin

**Affiliations:** 1 Department of Human Genetics, McGill University, Montreal, H3A 1B1, Canada; 2 Xceed Molecular, Toronto, M9W 1B3, Canada; 3 The Research Institute of the McGill University Health Centre, Montréal, H3G 1A4, Canada; 4 Centre de Recherche du Centre hospitalier de l'Université de Montréal/Institut du cancer de Montréal, Montréal, H2L 4M1, Canada; 5 Département de Médicine, Université de Montréal, Montréal, H3C 3J7, Canada; 6 Département de Obstétrique et Gynecologie, Division of Gynecologic Oncology, Université de Montréal, Montreal, Canada; 7 Department of Medicine, McGill University, Montreal, H3G 1A4, Canada

## Abstract

**Background:**

As gene expression signatures may serve as biomarkers, there is a need to develop technologies based on mRNA expression patterns that are adaptable for translational research. Xceed Molecular has recently developed a Ziplex^® ^technology, that can assay for gene expression of a discrete number of genes as a focused array. The present study has evaluated the reproducibility of the Ziplex system as applied to ovarian cancer research of genes shown to exhibit distinct expression profiles initially assessed by Affymetrix GeneChip^® ^analyses.

**Methods:**

The new chemiluminescence-based Ziplex^® ^gene expression array technology was evaluated for the expression of 93 genes selected based on their Affymetrix GeneChip^® ^profiles as applied to ovarian cancer research. Probe design was based on the Affymetrix target sequence that favors the 3' UTR of transcripts in order to maximize reproducibility across platforms. Gene expression analysis was performed using the Ziplex Automated Workstation. Statistical analyses were performed to evaluate reproducibility of both the magnitude of expression and differences between normal and tumor samples by correlation analyses, fold change differences and statistical significance testing.

**Results:**

Expressions of 82 of 93 (88.2%) genes were highly correlated (p < 0.01) in a comparison of the two platforms. Overall, 75 of 93 (80.6%) genes exhibited consistent results in normal versus tumor tissue comparisons for both platforms (p < 0.001). The fold change differences were concordant for 87 of 93 (94%) genes, where there was agreement between the platforms regarding statistical significance for 71 (76%) of 87 genes. There was a strong agreement between the two platforms as shown by comparisons of log_2 _fold differences of gene expression between tumor versus normal samples (R = 0.93) and by Bland-Altman analysis, where greater than 90% of expression values fell within the 95% limits of agreement.

**Conclusion:**

Overall concordance of gene expression patterns based on correlations, statistical significance between tumor and normal ovary data, and fold changes was consistent between the Ziplex and Affymetrix platforms. The reproducibility and ease-of-use of the technology suggests that the Ziplex array is a suitable platform for translational research.

## Background

During the last decade, the advent of high-throughput techniques such as DNA microarrays, has allowed investigators to interrogate the expression level of thousands of genes concurrently. Due to the heterogeneous nature of many cancers in terms of both their genetic and molecular origins and their response to treatment, individualizing patient treatment based on the expression levels of signature genes may impact favorably on patient management [1,2]. In ovarian cancer, discrete gene signatures have been determined from microarray analysis of ovarian cancer versus normal ovarian tissue [3-6], correlating gene expression profiles to survival or prognosis [7,8], studies of chemotherapy resistance [9,10], and functional studies such as chromosome transfer experiments [11,12]. Recent studies have focused on a biomarker approach [13], with specific prognostic markers being discovered by relating gene expression profiles to clinical variables [14-16]. In addition, there is a trend towards offering patient-tailored therapy, where expression profiles are related to key clinical features such as *TP53 *or *HER2 *status, surgical outcome and chemotherapy resistance [1,17].

A major challenge in translating promising mRNA-based expression biomarkers has been the reproducibility of results when adapting gene expression assays to alternative platforms that are specifically developed for clinical laboratories. Xceed Molecular has recently developed a multiplex gene expression assay technology termed the Ziplex^® ^Automated Workstation, designed to facilitate the expression analysis of a discrete number of genes (up to 120) specifically intended for clinical translational laboratories. The Ziplex array is essentially a three-dimensional array comprised of a microporous silicon matrix containing oligonucleotides probes mounted on a plastic tube. The probes are designed to overlap the target sequences of the probes used in large-scale gene expression array platforms from which the expression signature of interest was initially detected, such as the 3' UTR target sequences of the Affymetrix GeneChip^®^. However unlike most large-scale expression platforms, gene expression detection is by chemiluminescence. Recently, the Ziplex technology was compared to five other commercially available and well established gene expression profiling systems following the methods introduced by the MicroArray Quality Control (MAQC) consortium [18-20] and reported in a white paper by Xceed Molecular [21]. The original MAQC study (MAQC Consortium, 2006) was undertaken because of concerns about the reproducibility and cross-platform concordance between gene expression profiling platforms, such as microarrays and alternative quantitative platforms. By assessing the expression levels of the MAQC panel of 53 genes on universal RNA samples, it was determined that the reproducibility, repeatability and sensitivity of the Ziplex system were at least equivalent to that of other MAQC platforms [21].

There is a need to implement reliable gene expression technologies that are readily adaptable to clinical laboratories in order to screen individual or multiple gene expression profiles ("signature") identified by large-scale gene expression assays of cancer samples. Our ovarian cancer research group (as well as other independent groups) has identified specific gene expression profiles from mining Affymetrix GeneChip expression data illustrating the utility of this approach at identifying gene signature patterns associated with specific parameters of the disease [14,22]. Ovarian cancer specimens are typically large and exhibit less tumor heterogeneity and thus may be amenable to gene expression profiling in a reproducible way. However, until recently the gene expression technologies available that could easily be adapted to a clinical setting have been limited primarily by the expertise required to operate them. The recently developed Ziplex Automated Workstation offers a opportunity to develop RNA expression-based biomarkers that could readily be adapted to clinical settings as the 'all-in-one' technology appears to be relatively easy to use. However, this system has not been applied to ovarian cancer disease nor has its use been reported in human systems. In the present study we have evaluated the reproducibility of the Ziplex system using 93 genes, selected based on their expression profile as initially assessed by Affymetrix GeneChip microarray analyses from a number of ovarian cancer research studies from our group [6,14,22-26]. These include genes which are highly differentially expressed between ovarian tumor samples and normal ovary samples that were identified using both newer and older generation GeneChips [6,22,25,26]. In addition, to address the question of sensitivity, genes known to have a wide range of expression values were tested some of which show comparable values of expression between representative normal and ovarian tumor tissue samples but represent a broad range of expression values [25,26]. Other genes known to be relevant to ovarian cancer including tumor suppressor genes and oncogenes were included in the analysis. Selected highly differentially expressed genes from an independent microarray analysis of ovarian tumors compared to short term cultures of normal epithelial cells was also included [3]. In many cases, the level of gene expression identified by Affymetrix GeneChip analysis was independently validated by semi-quantitative RT-PCR, real-time RT-PCR, or Northern Blot analysis [6,14,22,24-26]. Expression assays were performed using RNA from serous ovarian tumors, short term cultures of normal ovarian surface epithelial cells, and four well characterized ovarian cancer cell lines which were selected based on their known expression profiles using Affymetrix microarray analyses. Comparisons were made between the Ziplex system and expression profiles generated using the U133A Affymetrix GeneChip platform. An important aspect of this study was that gene expression profiling of Ziplex system was performed in a blinded fashion where the sample content was not known to the immediate users. It is envisaged that both the nature of the candidates chosen and their range of gene expression will permit for a direct comment on the sensitivity, reproducibility and overall utility of the Ziplex array as a platform for gene expression array analysis for translational research.

## Methods

### Source of RNA

Total RNA was extracted with TRIzol reagent (Gibco/BRL, Life Technologies Inc., Grand Island, NY) from primary cultures of normal ovarian surface epithelial (NOSE) cells, frozen malignant serous ovarian tumor (TOV) samples and epithelial ovarian cancer (EOC) cell lines as described previously [27]. Additional File 1 provides a description of samples used in the expression analyses.

The NOSE and TOV samples were attained from the study participants at the Centre de recherche du Centre hospitalier de l'Université de Montréal – Hôpital Hotel-Dieu and Institut du cancer de Montréal with signed informed consent as part of the tissue and clinical banking activities of the Banque de tissus et de données of the Réseau de recherche sur le cancer of the Fonds de la Recherche en Santé du Québec (FRSQ). The study was granted ethical approval from the Research Ethics Boards of the participating research institutes.

### Ziplex array and probe design

The 93 genes used for assessing the reproducibility of the Ziplex array are shown in Table 1. The criteria for gene selection were: genes exhibiting statistically significant differential expression between NOSE and TOV samples as assessed by Affymetrix U133A microarray analysis; genes exhibiting a range of expression values (nominally low, medium or high) based on Affymetrix U133A microarray analysis, in order to assess sensitivity; genes exhibiting differential expression profiles based on older generation Affymetrix GeneChips (Hs 6000 [6] and Hu 6800 [23]); and genes known or suspected to play a role in ovarian cancer (Table 1). Initial selection criteria for genes in their original study included individual two-way comparisons [25,26], fold-differences [6,23], and fold change analysis using SAM (Significance Analysis of Microarrays) [3] between TOV and NOSE groups. Some genes were selected based on their low, mid or high range of expression values that did not necessarily exhibit statistically significant differences between TOV and NOSE groups.

**Table 1:** Selection Criteria of Genes Assayed by Ziplex Technology

**Selection Criteria Categories**	**Affymetrix U133A Probe Set**	GeneID*	**Gene Name**	**Reference**
**A: Differentially expressed genes based on Affymetrix U133A analysis**	208782_at	11167	*FSTL1*	25
	213069_at	57493	*HEG1*	25
	218729_at	56925	*LXN*	25
	202620_s_at	5352	*PLOD2*	25
	217811_at	51714	*SELT*	25
	213338_at	25907	*TMEM158*	25
	203282_at	2632	*GBE1*	25
	204846_at	1356	*CP*	25
	221884_at	2122	*EVI1*	25
	202310_s_at	1277	*COL1A1*	26
	201508_at	3487	*IGFBP4*	26
	200654_at	5034	*P4HB*	26
	212372_at	4628	*MYH10*	26
	216598_s_at	6347	*CCL2*	26
	208626_s_at	10493	*VAT1*	26
	41220_at	10801	*SEPT9*	26
	208789_at	284119	*PTRF*	26
	206295_at	3606	*IL18*	22
	202859_x_at	3576	*IL8*	22
	209969_s_at	6772	*STAT1*	22
	209846_s_at	11118	*BTN3A2*	22
	220327_at	389136	*VGLL3*	11
	203180_at	220	*ALDH1A3*	26
	204338_s_at	5999	*RGS4*	26
	204879_at	10630	*PDPN*	26
	207510_at	623	*BDKRB1*	26
	208131_s_at	5740	*PTGIS*	26
	211430_s_at	3500	*IGHG1*	26
	216834_at	5996	*RGS1*	26
	266_s_at	100133941	*CD24*	26
	213994_s_at	10418	*SPON1*	26
	221671_x_at	3514	*IGKC*	26
**B: Genes exhibiting a range of expression values based on Affymetrix U133A analysis**	218304_s_at	114885	*OSBPL11*	25
	219295_s_at	26577	*PCOLCE2*	25
	205329_s_at	8723	*SNX4*	25
	219036_at	80321	*CEP70*	25
	218926_at	55892	*MYNN*	25
	208836_at	483	*ATP1B3*	25
	204992_s_at	5217	*PFN2*	25
	214143_x_at	6152	*RPL24*	25
	208691_at	7037	*TFRC*	25
	203002_at	51421	*AMOTL2*	25
	221492_s_at	64422	*ATG3*	25
	218286_s_at	9616	*RNF7*	25
	212058_at	23350	*SR140*	25
	201519_at	9868	*TOMM70A*	25
	209933_s_at	11314	*CD300A*	26
	219184_x_at	29928	*TIMM22*	26
	204683_at	3384	*ICAM2*	26
	212529_at	124801	*LSM12*	26
	211899_s_at	9618	*TRAF4*	26
	218014_at	79902	*NUP85*	26
	200816_s_at	5048	*PAFAH1B1*	26
	202395_at	4905	*NSF*	26
	201388_at	5709	*PSMD3*	26
	220975_s_at	114897	*C1QTNF1*	26
	210561_s_at	26118	*WSB1*	26
	202856_s_at	9123	*SLC16A3*	26
	212279_at	27346	*TMEM97*	26
	37408_at	9902	*MRC2*	26
	201140_s_at	5878	*RAB5C*	26
	214218_s_at	7503	*XIST*	24
	200600_at	4478	*MSN*	24
	201136_at	5355	*PLP2*	24
**C: Genes exhibiting differential expression profiles based on older generation Affymetrix GeneChips (Hs 6000 (6), Hu 6800 (22))**	202431_s_at	4609	*MYC*	6
	203752_s_at	3727	*JUND*	6
	205009_at	7031	*TFF1*	6
	205067_at	3553	*IL1B*	6
	200807_s_at	3329	*HSPD1*	6
	203139_at	1612	*DAPK1*	6
	200886_s_at	5223	*PGAM1*	6
	203083_at	7058	*THBS2*	6
	202284_s_at	1026	*CDKN1A*	6
	212667_at	6678	*SPARC*	6
	202627_s_at	5054	*SERPINE1*	6
	203382_s_at	348	*APOE*	6
	211300_s_at	7157	*TP53*	6
	200953_s_at	894	*CCND2*	6
	201700_at	896	*CCND3*	6
	205881_at	7625	*ZNF74*	23
	207081_s_at	5297	*PI4KA*	23
	205576_at	3053	*SERPIND1*	23
	203412_at	8216	*LZTR1*	23
	206184_at	1399	*CRKL*	23

**D: Known oncogenes and tumour U133A analysis suppressor genes relevant to ovarian cancer biology**	203132_at	5925	*RB1*	
	204531_s_at	672	*BRCA1*	
	214727_at	675	*BRCA2*	
	202520_s_at	4292	*MLH1*	
	216836_s_at	2064	*ERBB2*	
	204009_s_at	3845	*KRAS*	
	206044_s_at	673	*BRAF*	
	209421_at	4436	*MSH2*	
	211450_s_at	2956	*MSH6*	

*GeneID (gene identification number) is based on the nomenclature used in the Entrez Gene database available through the National Center for Biotechnology Information (NCBI)

.

The Ziplex array or TipChip is a three-dimensional array comprised of a microporous silicon matrix containing oligonucleotide probes that is mounted on a plastic tube. Each probe was spotted in triplicate. In order to replicate gene expression assays derived from the Affymetrix GeneChip analysis, probe set design was based on the Affymetrix U133A probe set target sequences for the selected gene (refer to Table 1). Gene names were assigned using UniGene ID Build 215 (17 August 2008). To improve accuracy of probe design, and to account for variation of probe hybridization, up to three probes were designed for each gene. From this exercise, a single probe was chosen to provide the most reliable and consistent quantification of gene expression. Gene accession numbers corresponding to the Affymetrix probe set sequences for each gene were verified by BLAST alignment searches of the NCBI Transcript Reference Sequences (RefSeq) database . Array Designer (Premier Biosoft, Palo Alto, CA) was used to generate three probes from each verified RefSeq transcript that were between 35 to 50 bases in length (median 46 base pairs), exhibited a melting temperature of approximately 70°C, represent a maximum distance of 1,500 base pairs from the from 3' end of the transcript, and exhibited minimal homology to non-target RefSeq sequences. Using this approach it was possible to design three probes for 92 of the 93 selected genes: *APOE *was represented by only two probes. For the 93 genes analyzed, the median distance from the 3' end was 263 bases, whereas less than 12% of the probes were more than 600 bases from the 3' end. Ten probes were also designed for genes that were not expected to vary significantly between TOV and NOSE samples based on approximately equal expression in the two sample types and relatively low coefficients of variation (18 to 20%) as assessed by Affymetrix U133A microarray analysis of the samples; such probes were potential normalization controls. Based on standard quality control measures of the manufacturer, three probes representing *ACTB*, *GAPDH*, and *UBC *and a set of standard control probes, including a set of 5' end biased probes for *RPL4, POLR2A, ACTB, GAPDH *and *ACADVL *were printed on each array for data normalization and quality assessment. The probes were printed on two separate TipChip arrays.

### Hybridization and raw data collection

Total RNA from NOSE and TOV samples and the four EOC cell lines were prepared as described above and provided to Xceed Molecular for hybridization and data collection in a blinded manner. RNA quality (RNA integrity number (RIN)) using the Agilent 2100 Bioanalyzer Nano, total RNA assay was assessed for each sample (Additional File 1). For each sample, approximately 500 ng of RNA was amplified and labeled with the Illumina^® ^TotalPrep™ RNA Amplification Kit (Ambion, Applied Biosystems Canada, Streetsville, ON, CANADA). Although sample MG0026 (TOV-1150G) had a low RIN number, it was carried through the study. Sample MG0001 (TOV-21G) had no detectable RIN number and MG0013 (NOV-1181) failed to produce amplified RNA. Neither of these samples were carried through the study. Five μg of the resulting biotin-labeled amplified RNA was hybridized on each TipChip. The target molecules were biotin labeled, and an HRP-streptavidin complex was used for imaging of bound targets by chemiluminescence. Hybridization, washing, chemiluminescent imaging and data collection were automatically performed by the Ziplex Workstation (Xceed Molecular, Toronto, ON, Canada).

### Data normalization

The mean ratio of the intensities of the replicate probes that were printed on both of the ovarian cancer arrays were used to scale the data between the two TipChip arrays hybridized with each sample. The mean scaling factor for the 27 samples was 1.03 with a maximum of 1.23. The coefficients of variation (CV) across 27 samples and the expression differences between NOSE and TOV samples was calculated from the raw data for each of the 10 genes included on the arrays as potential normalization genes (Additional File 2). The geometric means of the signals for probes for *PARK7*, *PI4KB*, *TBCB*, and *UBC *with small CVs (mean of 25%) and insignificant differences between NOSE and TOV (p > 0.48) were used to normalize the data (refer to Additional File 2 for all normalization gene results). The data were analyzed with and without normalization.

### Selection of optimal probe design

The hybridization intensities of the replicate probes designed for each gene for the 27 samples were compared to choose a single probe per gene with optimal performance. This assessment was based on signal intensity (well above the noise level and within the dynamic range of the system), minimum distance from the 3' end of the target sequence and correlation between different probe designs. Minimum distance from the 3' end is a consideration since the RNA sample preparation process is somewhat biased to the 3' end of the transcripts. The signals for probes for the same target should vary proportionally between different samples if both probes bind to and only to the nominal target. Good correlation between different Ziplex probe designs for genes in the RefSeq database, as well as good correlation with the Affymetrix data and discrimination between sample types, infers that probes bind to the intended target sequences. Data from the chosen probe was used for all subsequent analysis. Correlations of signal intensities for pairs of probes for the same genes are presented in Additional File 3.

### Comparative analysis of Ziplex and Affymetrix data

Correlations between Ziplex and Affymetrix array datasets were calculated. The Affymetrix U133A data was previously derived from RNA expression analysis of the NOSE and TOV samples and EOC cell lines. Hybridization and scanning was performed at the McGill University and Genome Quebec Innovation Centre . MAS5.0 software (Affymetrix^® ^Microarray Suite) was used to quantify gene expression levels. Data was normalized by multiplying the raw value for an individual probe set (n = 22,216) by 100 and dividing by the mean of the raw expression values for the given sample data set, as described previously [23,28]. Affymetrix and Ziplex data were matched by gene, and correlations (p < 0.01, using values only of greater than 4) and a graphical representation was determined using Mathematica (Version 6.03) software (Wolfram Research, Inc., Champaign, IL, USA). Mean signal intensity values were log_2 _transformed and compared between NOSE and TOV data using a Welch Rank Sum Test, for both Affymetrix microarray and Ziplex array data. A p-value of less than 0.001 was used as the significance level.

Composition of mean-difference plots followed the method of Bland and Altman [29]. Briefly, the mean of the log_2 _fold change and the difference between the log_2 _fold change for the platforms under comparison were calculated and plotted. The 95% limits of agreement were calculated as follows: log_2 _fold change difference ± 1.96 × standard deviation of the log_2 _fold change difference.

### Quality control of Ziplex array data

The percent CVs were greater for probes with signals below 30. The overall median of the median probe percent CV was 4.7%. The median of the median percent CVs was 4.4% for probes with median intensities greater than 30, and 8.0% for probes with median CVs less than or equal to 30. The signal to noise (SNR) values is the average of the ratios for the net signals of the replicate spots to the standard deviation of the pixel values used to evaluate background levels (an image noise estimate). Average SNR ranged from -0.3 to 32.8. The signal intensities and ratios of intensity signals derived from 3' and 5' probes are shown in Additional File 4. Sample MG0001, which included many high 3'/5' ratios, was not included for subsequent analysis. The 3'/5' signal intensity ratios correlated with the RIN numbers and 28 S/18 S ratios (Additional File 5), indicating that, as expected, amplified RNA fragment lengths vary according to the integrity of the total RNA sample.

## Results

### Correlation of Affymetrix U133A and Ziplex array expression profiles

**Figure 1:**
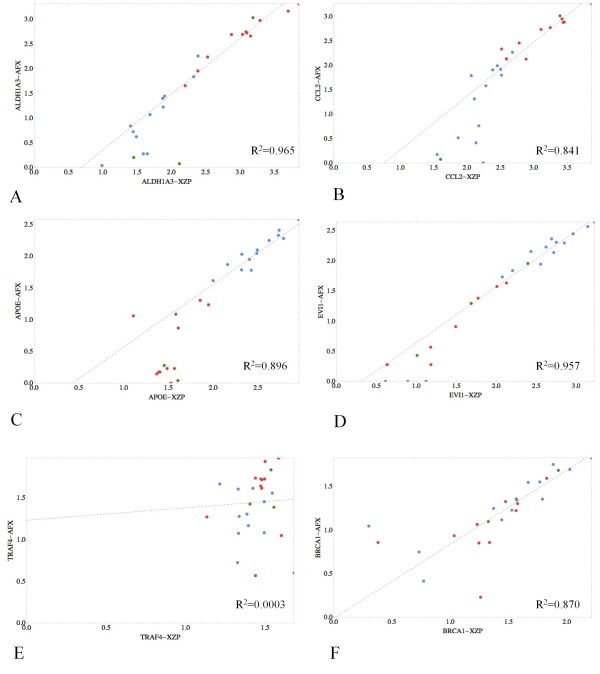
**Correlation plots of selected genes underexpressed in TOV (A, B), over-expressed in TOV (C, D) and showing low expression (E, F) across samples**. Xceed Ziplex (XZP) expression data is plotted on the x axis and Affymetrix (AFX) microarray data on the y axis. The EOC cell lines are indicated in green (n = 3), TOV samples in red (n = 12) and NOSE samples in blue (n = 11). Correlation coefficients are shown at the bottom right.

Normalized Affymetrix U133A and Ziplex gene expression data were matched by gene. For each gene expression platform, values less than 4 were considered to contribute to censoring bias and were not included in the correlation analysis. Correlations (log_10 _transformed) for paired gene expression data ranged from 0.0277 to 0.998, with an average correlation of 0.811 between Affymetrix and Ziplex gene expression data (Additional File 6). For a detailed summary of the correlation analysis, see also Additional File 7. The expression profiles of 82 of the 93 (88.2%) genes were significantly positively correlated (p < 0.01) in a comparison of the two platforms. As shown with the selected examples, genes exhibiting under-expression, such as *ALDH1A3 *and *CCL2*, or over-expression, such as *APOE *and *EVI1*, in the TOV samples relative to the NOSE samples by Affymetrix U133A microarray analysis also exhibited similar patterns of expression by Ziplex array (Figure 1). In contrast, *TRAF4 *expression was not correlated between the platforms (R^2 ^= 0.0003). However, both platforms yielded low expression values for this gene. Although gene expression at very low levels may be difficult to assay and can be affected by technical variability, a good correspondence between platforms can be achieved with specific probes, as shown in the comparison of the *BRCA1 *expression profiles (R^2 ^= 0.870) (Figure 1).

### Comparative analysis of fold changes of Affymetrix U133A and Ziplex array expression profiles

The fold change differences in gene expression were compared between the two platforms. There was a strong correspondence of gene expression patterns across the platforms when compared for each gene (Table 2). In terms of overall concordance of statistical significance between NOSE and TOV samples, there were consistent results for 75 of 93 genes by Affymetrix and Ziplex analysis (p < 0.001) by Welch rank sum test, in each platform. The fold change differences were concordant for 87 of 93 (94%) genes where there was agreement between the platforms regarding statistical significance for 71 (76%) of the 87 genes. The fold change differences were discordant for 6 genes, but the differences were statistically insignificant on both platforms for four of these genes. For example for the gene *SERPIND1*, there is no concordance in terms of fold change between the two platforms, but these fold change differences are not significant for either platform (p > 0.001). These results exemplifies that caution should be used when relying on fold change results alone. Notably, for two of the discordantly expressed genes (*MSH6 *and *TFF1*), the fold change differences were statistically significant (p < 0.001) only on the Ziplex platform but not for the Affymetrix platform.

**Table 2:** Comparison of mean signal intensity (SI) values for the 93 gene probes between NOSE and TOV samples

		Affymetrix U133A Array					Ziplex Automated Workstation					Platform Comparison	
Selection Criteria^1^	Gene Probe	NOSE mean SI (n = 11)	TOV mean SI (n = 12)	ratio (N/T)^2^	ratio (T/N)^2^	p-value^3^	NOSE mean SI (n = 11)	TOV mean SI (n = 12)	ratio (N/T)^2^	ratio (T/N)^2^	p-value^3^	significance based on p-value^3^	concordance based on ratio fold-change direction

A	RGS4	291	2	**181.2**	**0.01**	*<0.0001*	863	41	**21.1**	**0.05**	*<0.0001*	agree	concordance
C	SERPINE1	1912	12	**162.4**	**0.01**	*<0.0001*	1426	17	**82.2**	**0.01**	*<0.0001*	agree	concordance
A	PDPN	57	2	**23.9**	**0.04**	*0.0008*	100	35	**2.9**	**0.35**	0.0023	disagree	concordance
A	ALDH1A3	661	29	**22.6**	**0.04**	0.0020	1887	76	**24.8**	**0.04**	0.0051	agree	concordance
A	IL8	1353	69	**19.7**	**0.05**	0.0151	4465	231	**19.3**	**0.05**	0.0015	agree	concordance
A	PTGIS	1470	80	**18.4**	**0.05**	*<0.0001*	3474	184	**18.9**	**0.05**	*<0.0001*	agree	concordance
A	HEG1	923	66	**14.1**	**0.07**	*<0.0001*	3184	252	**12.6**	**0.08**	*<0.0001*	agree	concordance
A	TMEM158	461	33	**13.9**	**0.07**	*<0.0001*	869	46	**18.8**	**0.05**	*<0.0001*	agree	concordance
C	CDKN1A	598	53	**11.4**	**0.09**	*<0.0001*	385	63	**6.1**	**0.16**	*<0.0001*	agree	concordance
A	CCL2	570	54	**10.6**	**0.09**	*0.0010*	1923	207	**9.3**	**0.11**	*0.0001*	agree	concordance
A	LXN	731	73	**10.1**	**0.10**	*<0.0001*	926	124	**7.5**	**0.13**	*0.0002*	agree	concordance
C	SPARC	1037	108	**9.6**	**0.10**	*<0.0001*	2841	341	**8.3**	**0.12**	*<0.0001*	agree	concordance
C	IL1B	666	70	**9.6**	**0.10**	0.0247	1559	46	**34.0**	**0.03**	0.0035	agree	concordance
A	BDKRB1	152	18	**8.7**	**0.11**	*0.0004*	464	22	**21.0**	**0.05**	*<0.0001*	agree	concordance
B	SLC16A3	425	63	**6.8**	**0.15**	*<0.0001*	197	37	**5.3**	**0.19**	*<0.0001*	agree	concordance
A	FSTL1	1837	277	**6.6**	**0.15**	*<0.0001*	5293	732	**7.2**	**0.14**	*<0.0001*	agree	concordance
C	THBS2	846	135	**6.3**	**0.16**	*<0.0001*	668	105	**6.4**	**0.16**	*0.0009*	agree	concordance
A	IGFBP4	1484	238	**6.2**	**0.16**	*<0.0001*	692	122	**5.7**	**0.18**	*0.0001*	agree	concordance
A	PTRF	976	168	**5.8**	**0.17**	*<0.0001*	217	77	**2.8**	**0.35**	*<0.0001*	agree	concordance
A	GBE1	775	136	**5.7**	**0.18**	*<0.0001*	988	173	**5.7**	**0.17**	*<0.0001*	agree	concordance
A	PLOD2	654	123	**5.3**	**0.19**	*<0.0001*	926	132	**7.0**	**0.14**	*<0.0001*	agree	concordance
A	VAT1	874	175	**5.0**	**0.20**	*<0.0001*	255	78	**3.3**	**0.31**	*<0.0001*	agree	concordance
A	COL1A1	2940	614	**4.8**	**0.21**	*0.0001*	1502	289	**5.2**	**0.19**	*0.0003*	agree	concordance
C	CCND2	324	70	**4.7**	**0.21**	0.0127	481	117	**4.1**	**0.24**	0.0337	agree	concordance
A	SELT	558	148	**3.8**	**0.27**	*0.0010*	166	137	1.2	0.8	>0.05	disagree	concordance
B	C1QTNF1	169	48	**3.6**	**0.28**	*<0.0001*	30	3	**11.7**	**0.09**	*<0.0001*	agree	concordance
A	VGLL3	35	10	**3.5**	**0.29**	*<0.0001*	75	12	**6.1**	**0.16**	0.0015	disagree	concordance
C	PGAM1	1482	473	**3.1**	**0.32**	*<0.0001*	1603	504	**3.2**	**0.31**	*<0.0001*	agree	concordance
C	TP53	55	18	**3.0**	**0.33**	0.0178	197	226	0.9	1.1	>0.05	agree	discordance
B	MSN	746	250	**3.0**	**0.33**	*<0.0001*	818	354	**2.3**	**0.43**	*<0.0001*	agree	concordance
B	PSMD3	196	66	**3.0**	**0.34**	*<0.0001*	735	384	1.9	0.5	*<0.0001*	agree	concordance
B	WSB1	300	103	**2.9**	**0.34**	*0.0003*	313	155	**2.0**	**0.50**	*0.0006*	agree	concordance
B	MRC2	313	109	**2.9**	**0.35**	*<0.0001*	528	138	**3.8**	**0.26**	*<0.0001*	agree	concordance
A	MYH10	1113	420	**2.6**	**0.38**	*0.0006*	1096	464	**2.4**	**0.42**	0.0106	disagree	concordance
B	NSF	180	72	**2.5**	**0.40**	*<0.0001*	304	170	1.8	0.6	0.0023	disagree	concordance
A	P4HB	2276	917	**2.5**	**0.40**	*<0.0001*	4567	1553	**2.9**	**0.34**	*<0.0001*	agree	concordance
C	SERPIND1	7	3	**2.2**	**0.45**	>0.05	79	117	0.7	1.5	0.0363	agree	discordance
B	RAB5C	309	142	**2.2**	**0.46**	0.0106	132	61	**2.2**	**0.46**	*<0.0001*	disagree	concordance
B	PFN2	800	392	**2.0**	**0.49**	*<0.0001*	699	444	1.6	0.6	*0.0005*	agree	concordance
B	TRAF4	47	23	**2.0**	**0.50**	0.0363	30	27	1.1	0.9	>0.05	agree	concordance
B	LSM12	59	31	1.9	0.5	0.0023	53	36	1.5	0.7	0.0106	agree	concordance
B	PLP2	294	157	1.9	0.5	0.0051	270	190	1.4	0.7	0.0151	agree	concordance
B	PAFAH1B1	181	98	1.9	0.5	*0.0006*	556	387	1.4	0.7	0.0089	disagree	concordance
B	TIMM22	42	23	1.8	0.5	0.0392	126	82	1.5	0.6	*0.0001*	disagree	concordance
B	AMOTL2	308	173	1.8	0.6	0.0015	776	484	1.6	0.6	0.0113	agree	concordance
B	ATP1B3	668	386	1.7	0.6	*<0.0001*	832	449	1.9	0.5	0.0015	disagree	concordance
C	DAPK1	181	117	1.5	0.6	>0.05	186	146	1.3	0.8	>0.05	agree	concordance
B	TFRC	894	606	1.5	0.7	0.0089	386	216	1.8	0.6	0.0062	agree	concordance
B	ATG3	200	139	1.4	0.7	0.0106	342	319	1.1	0.9	>0.05	agree	concordance
B	RNF7	177	125	1.4	0.7	0.0178	54	63	0.9	1.2	>0.05	agree	concordance
A	IL18	21	16	1.4	0.7	0.0148	125	104	1.2	0.8	0.0210	agree	concordance
C	CRKL	38	28	1.4	0.7	>0.05	18	23	0.8	1.3	>0.05	agree	concordance
B	XIST	103	76	1.4	0.7	>0.05	256	378	0.7	1.5	>0.05	agree	discordance
C	PI4KA	59	44	1.4	0.7	0.0127	110	113	1.0	1.0	>0.05	agree	concordance
D	MSH6	62	47	1.3	0.8	>0.05	227	519	0.4	2.3	*0.0010*	disagree	discordance
C	LZTR1	82	69	1.2	0.8	>0.05	81	74	1.1	0.9	>0.05	agree	concordance
D	MLH1	171	150	1.1	0.9	>0.05	143	150	1.0	1.0	>0.05	agree	concordance
C	MYC	151	142	1.1	0.9	>0.05	119	212	0.6	1.8	>0.05	agree	discordance
B	PCOLCE2	22	21	1.0	1.0	>0.05	39	39	1.0	1.0	>0.05	agree	concordance
C	CCND3	136	139	1.0	1.0	>0.05	101	134	0.7	1.3	0.0127	agree	concordance
D	KRAS	157	162	1.0	1.0	>0.05	150	200	0.8	1.3	>0.05	agree	concordance
A	SEPT9	880	918	1.0	1.0	>0.05	543	394	1.4	0.7	>0.05	agree	concordance
D	RB1	67	73	0.9	1.1	>0.05	166	225	0.7	1.4	>0.05	agree	concordance
D	BRCA2	10	12	0.8	1.2	>0.05	15	23	0.6	1.6	0.0210	agree	concordance
B	SNX4	43	52	0.8	1.2	>0.05	199	339	0.6	1.7	0.0042	agree	concordance
A	BTN3A2	40	48	0.8	1.2	>0.05	89	173	0.5	1.9	*0.0005*	disagree	concordance
C	TFF1	12	16	0.7	1.4	>0.05	226	61	**3.7**	**0.3**	*<0.0001*	disagree	discordance
B	NUP85	71	101	0.7	1.4	>0.05	85	134	0.6	1.6	0.0028	agree	concordance
C	JUND	759	1181	0.6	1.6	>0.05	1725	2479	0.7	1.4	>0.05	agree	concordance
B	OSBPL11	46	74	0.6	1.6	0.0151	56	148	**0.4**	**2.6**	*<0.0001*	disagree	concordance
D	BRCA1	15	24	0.6	1.6	>0.05	27	40	0.7	1.5	>0.05	agree	concordance
B	SR140	144	243	0.6	1.7	0.0089	13	64	**0.2**	**5.0**	*<0.0001*	disagree	concordance
D	BRAF	27	46	0.6	1.7	0.0089	22	47	**0.5**	**2.1**	*<0.0001*	disagree	concordance
C	ZNF74	12	21	0.6	1.8	0.0042	16	44	**0.4**	**2.8**	*0.0002*	disagree	concordance
B	TOMM70A	212	383	0.6	1.8	*0.0004*	115	306	**0.4**	**2.7**	*<0.0001*	agree	concordance
B	RPL24	1895	3503	**0.5**	1.8	*0.0002*	1834	4179	**0.4**	**2.3**	*0.0003*	agree	concordance
C	HSPD1	899	1682	**0.5**	1.9	*0.0002*	461	1189	**0.4**	**2.6**	*0.0004*	agree	concordance
D	MSH2	27	53	**0.5**	2.0	0.0023	112	495	**0.2**	**4.4**	*<0.0001*	disagree	concordance
B	MYNN	27	55	**0.5**	**2.1**	*0.0001*	16	40	**0.4**	**2.5**	*0.0005*	agree	concordance
D	ERBB2	99	230	**0.4**	**2.3**	*0.0003*	50	142	**0.4**	**2.8**	*0.0002*	agree	concordance
B	ICAM2	14	34	**0.4**	**2.5**	0.0011	13	25	0.5	1.9	0.0089	agree	concordance
B	CEP70	23	59	**0.4**	**2.6**	*<0.0001*	56	182	**0.3**	**3.3**	*<0.0001*	agree	concordance
B	TMEM97	70	195	**0.4**	**2.8**	0.0015	51	140	**0.4**	**2.8**	*0.0004*	disagree	concordance
B	CD300A	11	36	**0.3**	**3.3**	*<0.0001*	4	36	**0.1**	**9.2**	*0.0006*	agree	concordance
A	STAT1	30	109	**0.3**	**3.6**	0.0127	48	110	**0.4**	**2.3**	0.0210	agree	concordance
A	EVI1	11	197	**0.06**	**17.5**	*<0.0001*	36	636	**0.06**	**17.5**	*<0.0001*	agree	concordance
C	APOE	7	126	**0.06**	**17.9**	*<0.0001*	39	326	**0.12**	**8.4**	*<0.0001*	agree	concordance
A	CP	7	295	**0.02**	**43.5**	*<0.0001*	33	972	**0.03**	**29.3**	*<0.0001*	agree	concordance
A	RGS1	2	112	**0.02**	**47.0**	*<0.0001*	3	169	**0.02**	**56.5**	*<0.0001*	agree	concordance
A	SPON1	5	271	**0.02**	**57.8**	*<0.0001*	6	257	**0.02**	**44.9**	*<0.0001*	agree	concordance
A	CD24	6	481	**0.01**	**77.2**	*<0.0001*	63	3697	**0.02**	**58.5**	*<0.0001*	agree	concordance
A	IGKC	7	991	**0.01**	**151.6**	*<0.0001*	27	873	**0.03**	**32.6**	*0.0008*	agree	concordance
A	IGHG1	3	1262	**0.003**	**374.3**	*<0.0001*	19	203	**0.10**	**10.5**	*<0.0001*	agree	concordance

^1^See Table 1 for description of categories of selection criteria. ^2^Fold change >2 or <0.5 (bold) between NOSE (N) and TOV (T) gene expression comparison. ^3^Welch Rank Sum Test p<0.001 (italics) difference between NOSE (N) and TOV (T).

**Figure 2:**
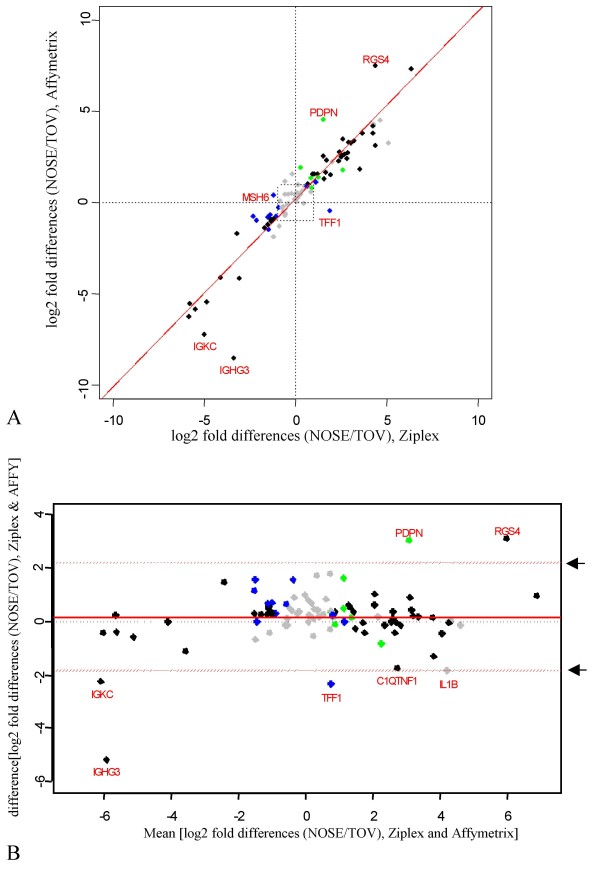
**Comparison of the fold change difference in expression between NOSE and TOV samples for the Ziplex and Affymetrix platforms**. **A**: The log_2 _fold change between the NOSE and TOV samples (mean NOSE signal intensity/mean TOV signal intensity) was calculated for the expression values of all 93 probes and plotted. Linear regression was performed resulting in the following model: log_2 _Affymetrix NOSE/TOV = 0.180098 + 1.0251794 log_2 _Ziplex NOSE/TOV with a Pearson's correlation coefficient (R) of 0.93. Probes that were not significant (p > 0.001 based on a Welch Rank Sum test) on either platform are indicated in grey, probes significant (p < 0.001 based on a Welch Rank Sum test) on both platforms are indicated in black, on only the Ziplex platform are indicated in blue and on only the Affymetrix platform in green. **B**: Bland-Altman plots for expression values of all probes. Values determined to be outliers are indicated in the mean-difference (of the log_2 _fold change values) plot. A difference in log_2 _fold change of 0 is indicated by a solid black line. The upper and lower 95% limits of agreement for the difference in log_2 _fold change are indicated by red dashed lines, and arrows on the right hand side. Expression values that fall outside of these lines are considered outliers and are identified by their gene name.

As shown in Figure 2A, there was a strong agreement between the two platforms as shown by comparisons of log_2 _fold differences of gene expression between TOV versus NOSE samples (R = 0.93) and by Bland-Altman analysis (Figure 2B), where the majority of probes exhibited expression profiles in comparative analyses that fell within the 95% limits of agreement. Both statistical methods of comparative analysis of log_2 _fold differences show minimal variance as the mean increases regardless of the direction of expression difference evaluated: genes selected based on over- or under-expression in TOV samples relative to NOSE samples. Although there were examples of expression differences which fell outside the 95% limits of agreement as observed in the Bland-Altman analysis such as for *RGSF4*, *PDPN*, *IGKC*, *IGHG1*, *C1QTNF1*, *TFF1 *and *IL1B *(Figure 2B), both the directionality and magnitude of TOV versus NOSE expression patterns were generally consistent (Figure 2A and Table 2).

## Discussion

The Ziplex array technology as applied to ovarian cancer research was capable of reproducing expression profiles of genes selected based on their Affymetrix GeneChip patterns. A high concordance of gene expression patterns was evident based on overall correlations, significance testing and fold-change comparisons derived from both platforms. The Ziplex array technology was validated by testing the expression of genes exhibiting not only significant differences in expression between normal tissues (NOSE) and ovarian cancer (TOV) samples but also the vast range in expression values exhibited by these samples using the Affymetrix microarray technology. Notable also is that comparisons were made between Affymetrix GeneChip data that was derived using MAS5 software rather than RMA analysis. We have routinely used MAS5 derived data in order to avoid potential skewing of low and high expression values which could occur with RMA treated data sets as this is more amenable to data sets of limited sample size [6,23,25,26,30]. MAS5 derived data also allows for exclusion of data that may represent ambiguous expression values as reflected in a reliability score based on comparison of hybridization to sets of probes representing matched and mismatch sequences complementary to the intended target RNA sequence. A recent study has re-evaluated the merits of using MAS5 data with detection call algorithms demonstrating its overall utility [31]. Our results are consistent with a previous study which had tested the analytical sensitivity, repeatability and differential expression of the Ziplex technology within a MAQC study framework [21]. As with all gene expression platforms, reproducibility is more variable within very low range of gene expression. Gene expression values in the low range across comparable groups would unlikely be developed as RNA expression biomarkers at the present time regardless of platform used. The MAQC study included a comparison of Xceed Molecular platform performance with at least three major gene expression platforms in current use in the research community, such as Affymetrix GeneChips, Agilent cDNA arrays, and real-time RT-PCR. The implementation of some of these various technology platforms in a clinical setting may require significant infrastructure which may be awkward to implement due to the level of expertise involved. In some cases, costs may also be prohibitive but this should diminish over time with increase in usage in clinical settings. It is also not clear that expression biomarkers are readily adaptable to all cancer types as this requires sufficient clinical specimens to extract amounts of good quality RNA for RNA biomarker screening to succeed. Tumor heterogeneity is also an issue. The large size and largely tumor cell composition of ovarian cancer specimens may render this disease more readily amenable to the development and implementation of RNA biomarker screening strategies in order to improve health care of ovarian cancer patients. The ease with which to use the Ziplex Automated Workstation focus array and the fact that it appears to perform overall as well as highly sensitive gene expression technologies including real-time RT-PCR, suggests that this new platform might be amenable to translational research of gene expression-based biomarkers for ovarian cancer initially identified from established large-scale gene expression platforms.

Data normalization of gene expression values is a subject of intense study and is a major consideration when moving from one technology platform to another [4,5]. In this study, data normalization of the Ziplex data was achieved by using the expression values derived from seven genes, each of which had low CV values across all samples tested. Since the input quantity of amplified RNA was equivalent for all Ziplex arrays, raw data could also have been used in our analysis. A statistical analysis based on correlations and fold-changes found negligible differences between raw and normalized data (not shown). Affymetrix GeneChip and Ziplex systems also differ in a number of technical ways that may affect the determination of gene expression. Affymetrix probe design is based on 11 oligonucleotide probes, 25 base pairs in size, within a target sequence of several hundred base pairs. The gene expression value is based on the median of the measured signal from the 11 probes. The probe design for the Ziplex system is based on oligonucleotide probes ranging from 35 to 50 bases. In this study three probes were designed and tested for each target gene and a single optimal probe was chosen. The visualization system for gene expression differs for both platforms where expression using the Ziplex array is measured by chemiluminescence, whereas fluorescence is used for the Affymetrix GeneChip. In spite of these differences, our findings along with an independent assessment of the Ziplex system [21] indicated a high degree of correspondence in expression profiles generated across both platforms. The overall findings are not surprising given that the probe design was intentionally targeted to similar 3'UTR sequences for the tested gene. Thus, the overall reproducibility of expression profiles along with the possibility of using raw data would be an attractive feature of applying the Ziplex system to validated biomarkers that were discovered using the Affymetrix platform.

The expression patterns of many of the tested genes were previously validated by an independent technique from our research group. RT-PCR analyses of ovarian cancer samples validated gene expression profiles of *TMEM158*, *GBE1 *and *HEG1 *from a chromosome 3 transcriptome analysis [25] and *IGFBP4*, *PTRF *and *C1QTNF1 *from a chromosome 17 transcriptome analysis [26]. The Ziplex platform also revealed over-expression of genes (*ZNF74*, *PIK4CA*, *SERPIND1*, *LZTR1 *and *CRKL*) associated with a chromosome 22q11 amplicon found in the OV90 EOC cell line and initially characterized by earlier generation Affymetrix expression microarrays and validated by RT-PCR and Northern blot analysis [23]. Differential expression of *SPARC*, a tumor suppressor gene implicated in ovarian cancer, has been shown to give consistent expression profiles in EOC cell lines and samples across a number of Affymetrix GeneChip^® ^platforms and by RT-PCR from our group and others [6,30,32]. This indicates the utility of using older generation Affymetrix GeneChip data where good concordance can be observed with historical data and the accuracy of the earlier generation GeneChips has been evaluated by alternative techniques in the literature [6,23]. This is an important consideration particularly given the large number of historical data sets that are available for further mining of potential gene expression biomarkers. Northern blot analysis has validated expression of *MYC*, *HSPD1*, *TP53 *and *PGAM1 *which were initially found to be differentially expressed in our EOC cell lines by the prototype Affymetrix GeneChip [6]. Concordance of gene expression was also evident from the 10 genes (see Table 1) selected based on an Affymetrix U133A microarray analysis of TOV samples and short term cultures of NOSE samples reported by an independent group [3]. *BTF4 *is a potential prognostic marker for ovarian cancer and was originally identified by Affymetrix microarray technology and then validated by real-time RT-PCR analysis [14]. Assaying the expression of *BTF4 *in clinical specimens is of particular interest because at the time of study there was no available antibody, illustrating the need for a reliable and accurate quantitative gene expression platform for RNA molecular markers.

## Conclusion

It is becoming increasingly apparent that expression signatures involving multiple genes can be correlated with various clinical parameters of disease, and in turn that these signatures could be used as biomarkers [4,5]. Although the expression signatures are gleaned from the statistical analyses of transcriptomes from genome-wide expression analyses, such as with use of Affymetrix GeneChip, the use of such arrays requires technical expertise and infrastructure that is not at the present time readily adaptable to clinical laboratories. In this study we have shown the concordance of the expression signatures derived from Affymetrix microarray analysis by the Ziplex array technology, suggesting that it is amenable for translational research of expression signature biomarkers for ovarian cancer.

## List of abbreviations used

RNA: ribonucleic acid; mRNA: messenger ribonucleic acid; UTR: untranslated region; R: correlation coefficient; MAQC: MicroArray Quality Control; RT-PCR: reverse transcription polymerase chain reaction; NOSE cells: normal ovarian surface epithelial cells; TOV: ovarian tumor; EOC: epithelial ovarian cancer; BLAST: Basic Local Alignment Search Tool; NCBI: National Centre for Biotechnology Information; RIN: RNA integrity number; HRP: horseradish peroxidase; SNR: signal to noise ratio; SI: signal intensity.

## Competing interests

DW, FY, AD and DE are employees of Xceed Molecular.

## Authors' contributions

MQ contributed to candidate gene selection for the study, sample selection, performed data analysis (correlations), results interpretation and wrote the majority of the paper. AMMM, DP, SA, AB and PW aiding in selecting candidate genes, preliminary results analysis and review of the paper draft. DW and FY performed sample quality control, RNA amplification and hybridization at Xceed Molecular. AD performed statistical analysis and aided with the writing of the draft. DE designed Ziplex probes, performed preliminary data analysis and contributed to the writing of the draft. PT and DE conceptualized the project, and aided in writing the initial draft. PT was the project leader. All authors read and approved the final manuscript.

## Supplementary Material

Additional file 1 **Sample description**. RNA samples used in the expression analyses. Click here for file

Additional file 2 **Genes for normalization**. Differential expression between NOSE and TOV in the raw data (log_2 _ratios, and T-test). Click here for file

Additional file 3 **Correlations between different probe designs for the same target gene**. Three different probes were designed and tested for each of the target genes, except for one of the genes (APOE) for which there were two designs. Each row of plots contains correlations between probes for a given gene. The accession numbers and gene symbols are indicated on the plots. Plots with linear scales are shown on the left, and plots with log10scales are shown on the right. The probes are identified in the axis labels with an Xceed part number and the gene symbol. The distance of each probe from the 3' end of the sequence corresponding to the accession number is shown after the colon in the axis labels. The colors used to plot the data for each sample are: NOSE samples – blue, TOV samples – red, cell line samples – green. Low intensity probes are plotted with open symbols. Click here for file

Additional file 4 **Signal intensities and 3'/5' ratios for all ten 5' control probes on duplicate chips**. 3', 5' signial intensities and 3'/5' ratios for each sample, for the genes RPL4, POL2RA, ACTB, GAPD and ACADVL2. Click here for file

Additional file 5 **RNA quality control**. Correlation between the geometric mean of seven 3'/5' control probe ratios and RIN number or 28 S/18 S ratios. Samples MG0001 (TOV-21G) and MG0026 (NOSE-1181) are not included. Click here for file

Additional file 6 **Correlations between Affymetrix U133A and Xceed Ziplex data**. Correlation graphs plotted for all 93 study genes, organized alphabetically. TOV samples are shaded red, NOSE blue and cell lines are indicated in green. Click here for file

Additional file 7 **Correlation analysis of Ziplex versus Affymetrix gene expression data**. Correlation analysis for all genes including p-value and R-squared. Click here for file
