# Supplementary figures and images for: The chemiluminescence based Ziplex® automated workstation focus array reproduces ovarian cancer Affymetrix GeneChip® expression profiles
Source: J Transl Med. 2009 Jul 6;7:55. doi: 10.1186/1479-5876-7-55 (PMC2724495; doi:10.1186/1479-5876-7-55)

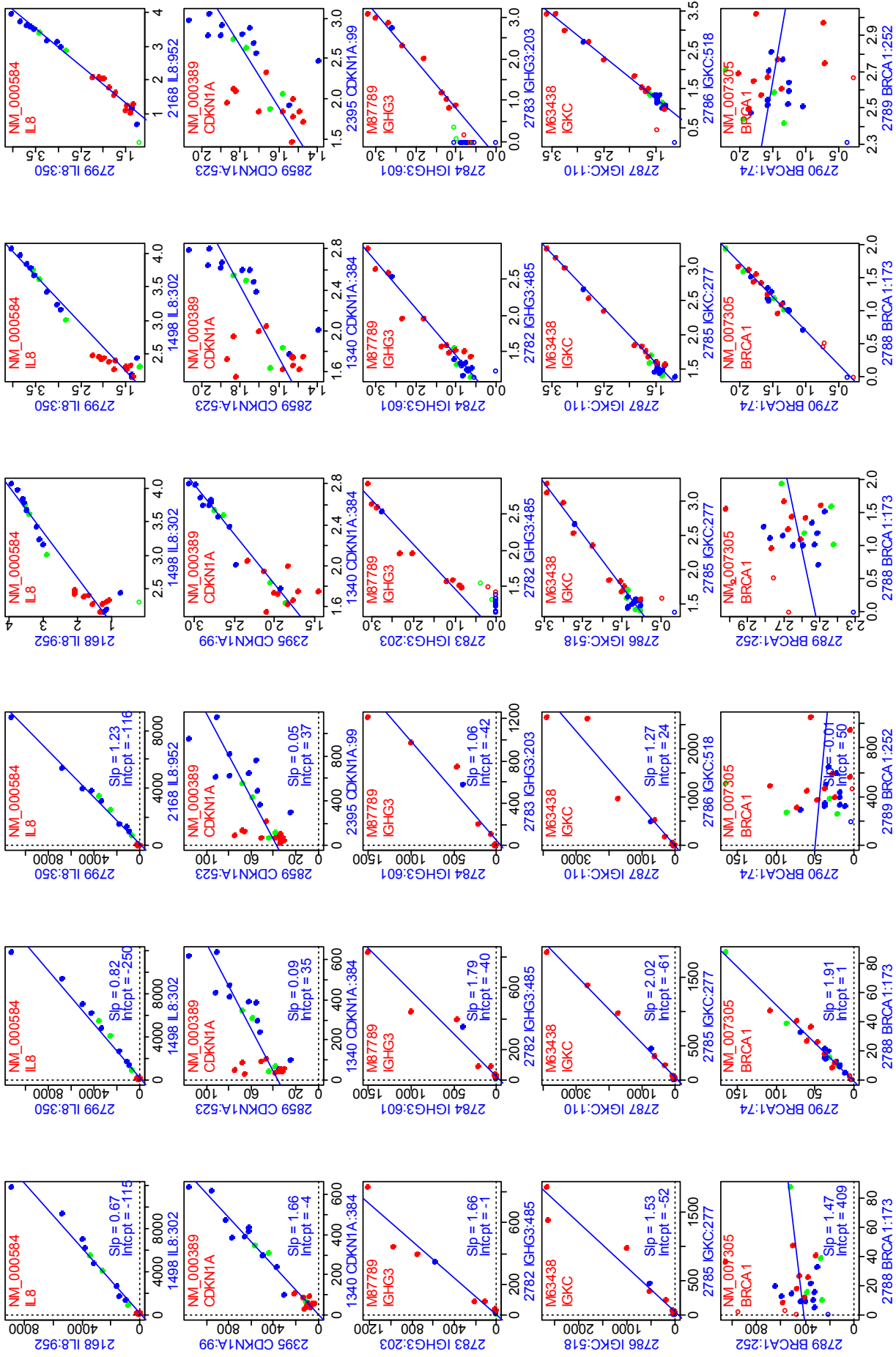

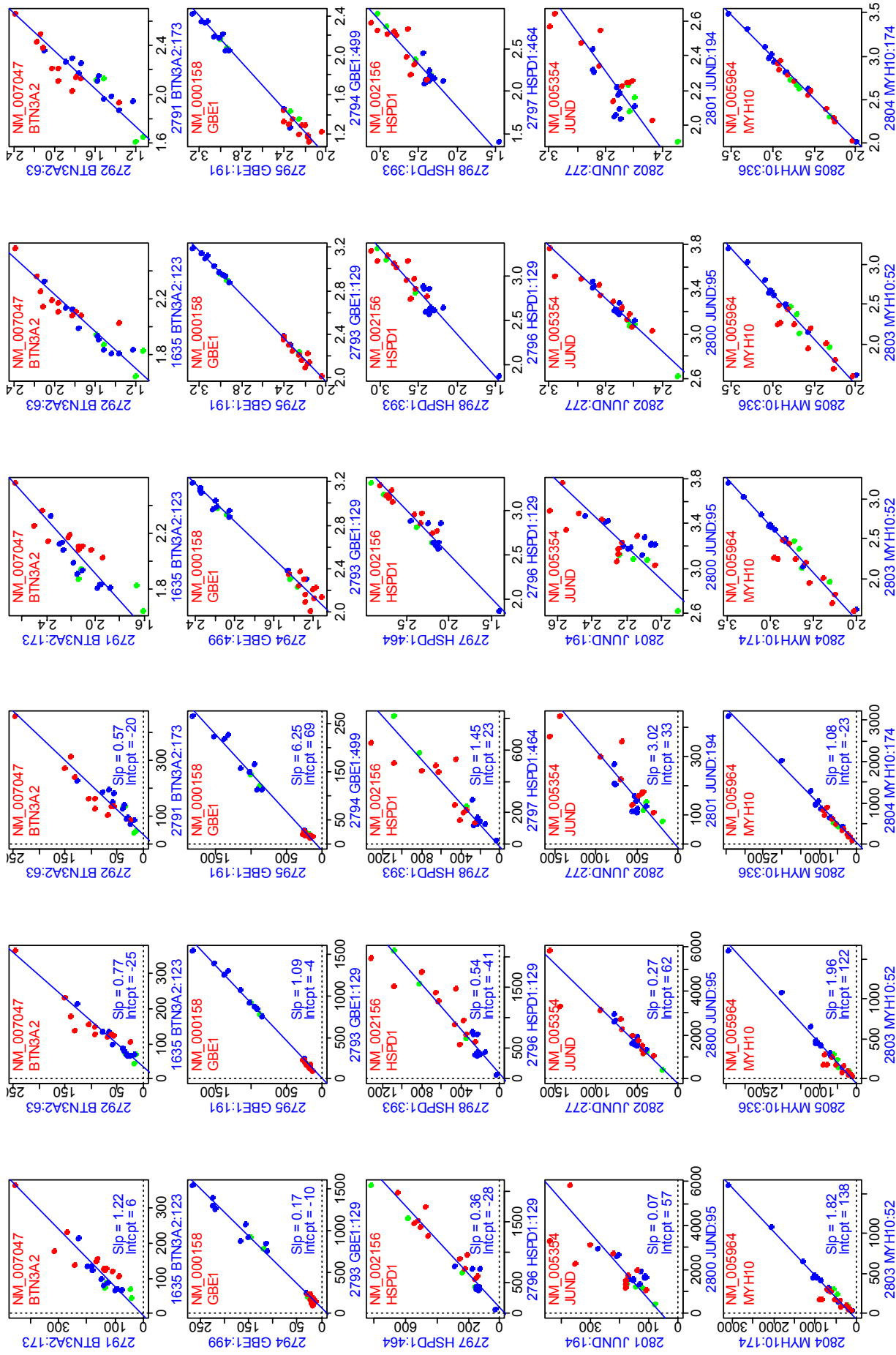

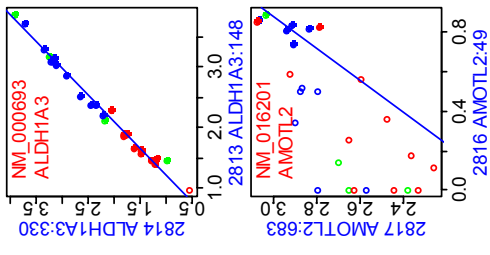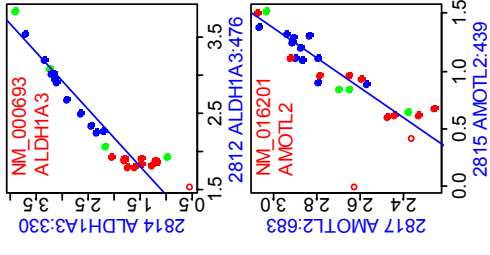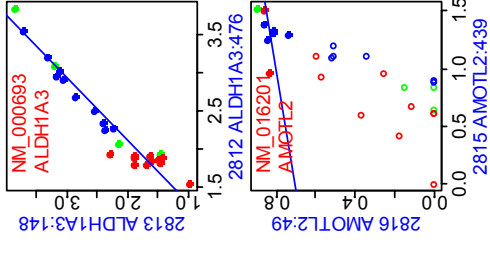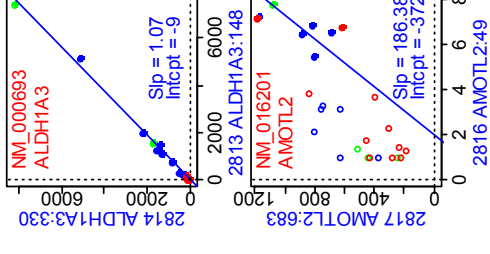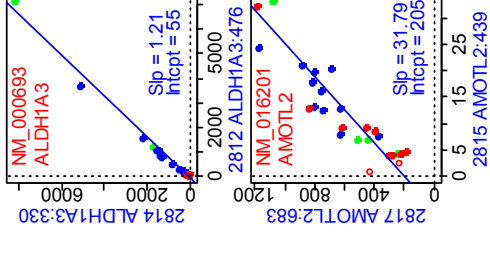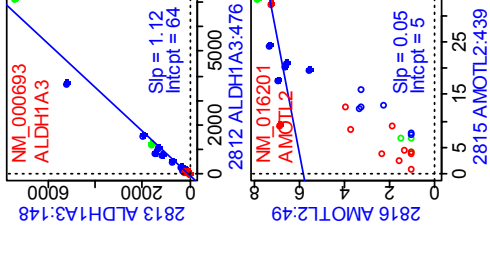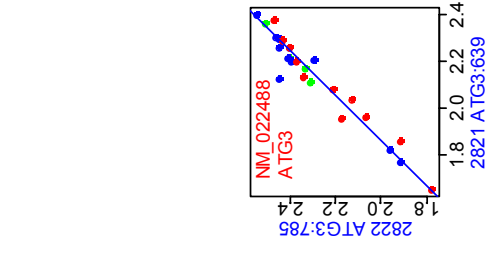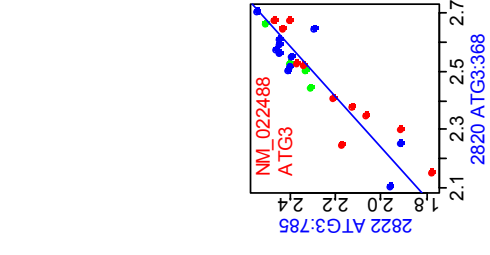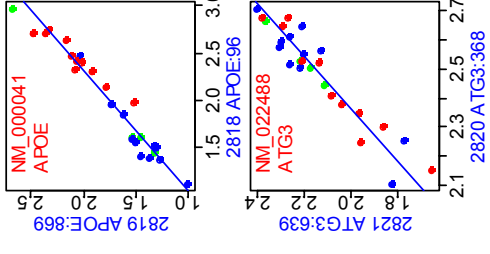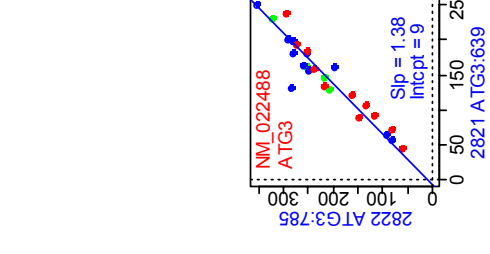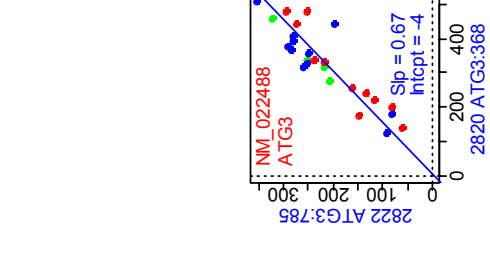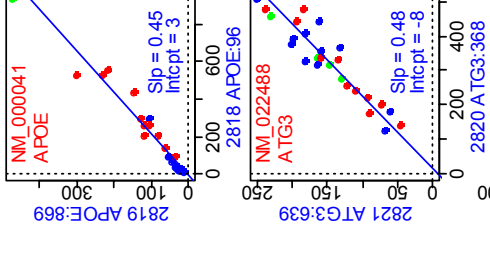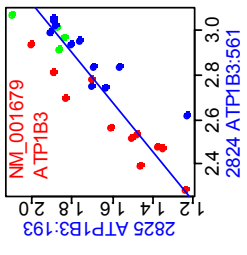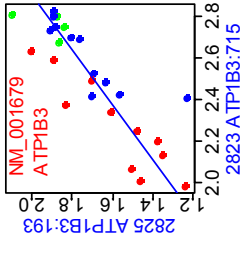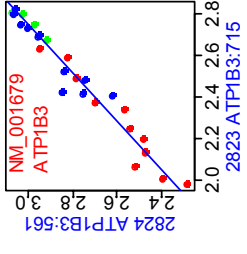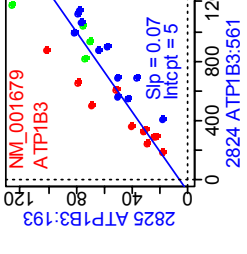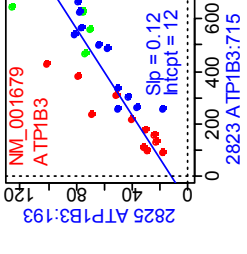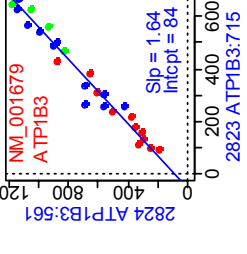

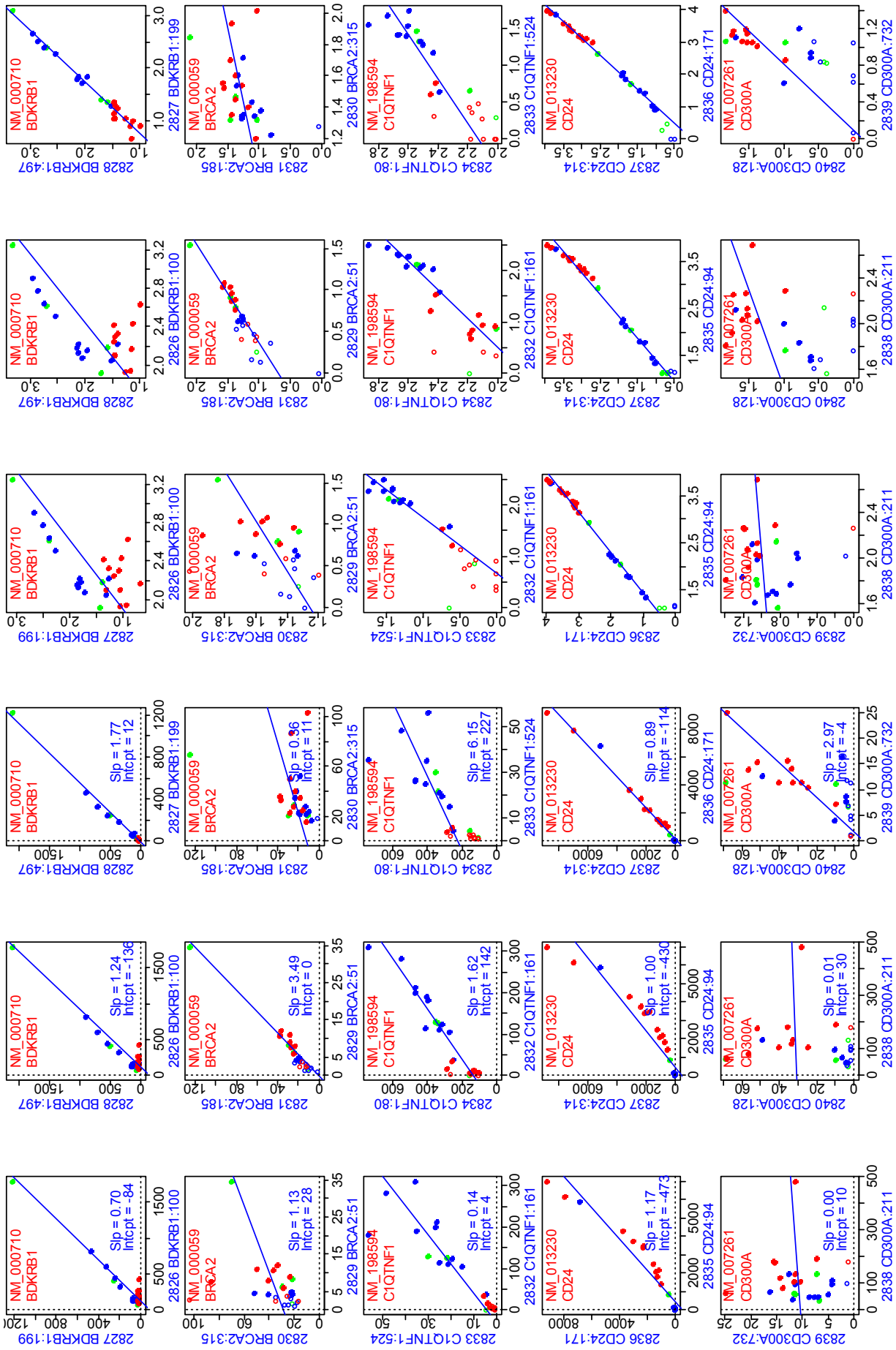

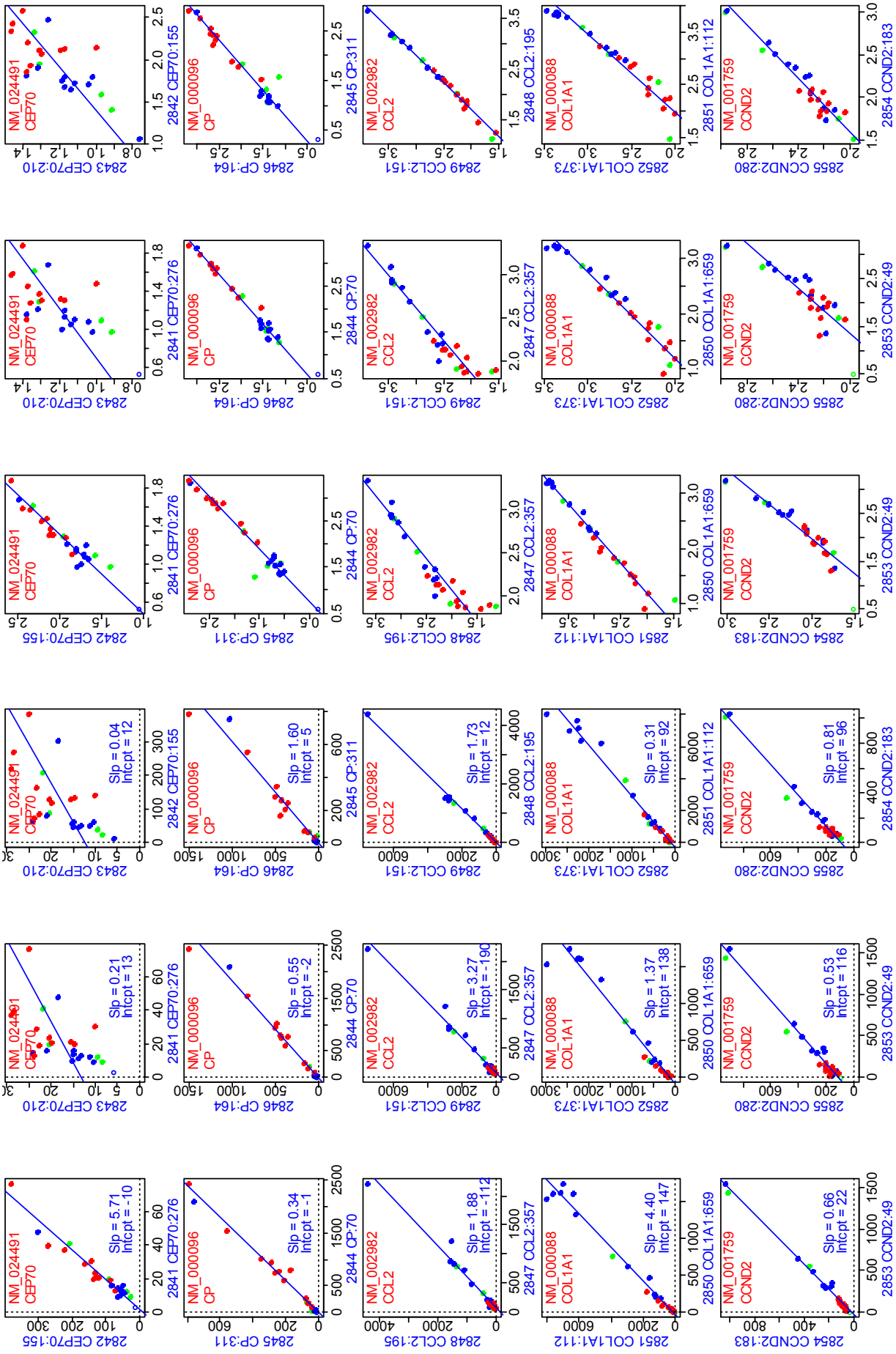

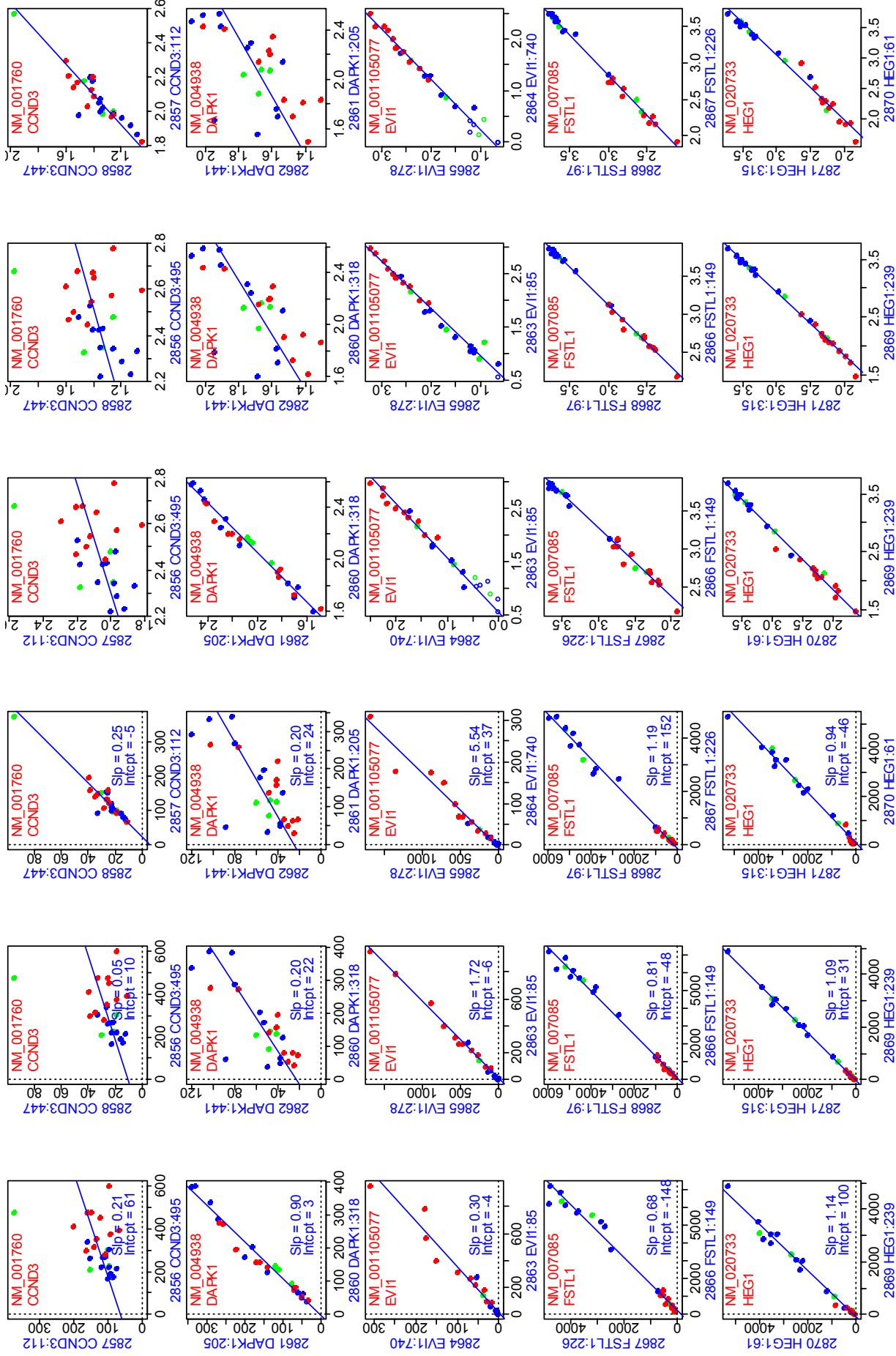

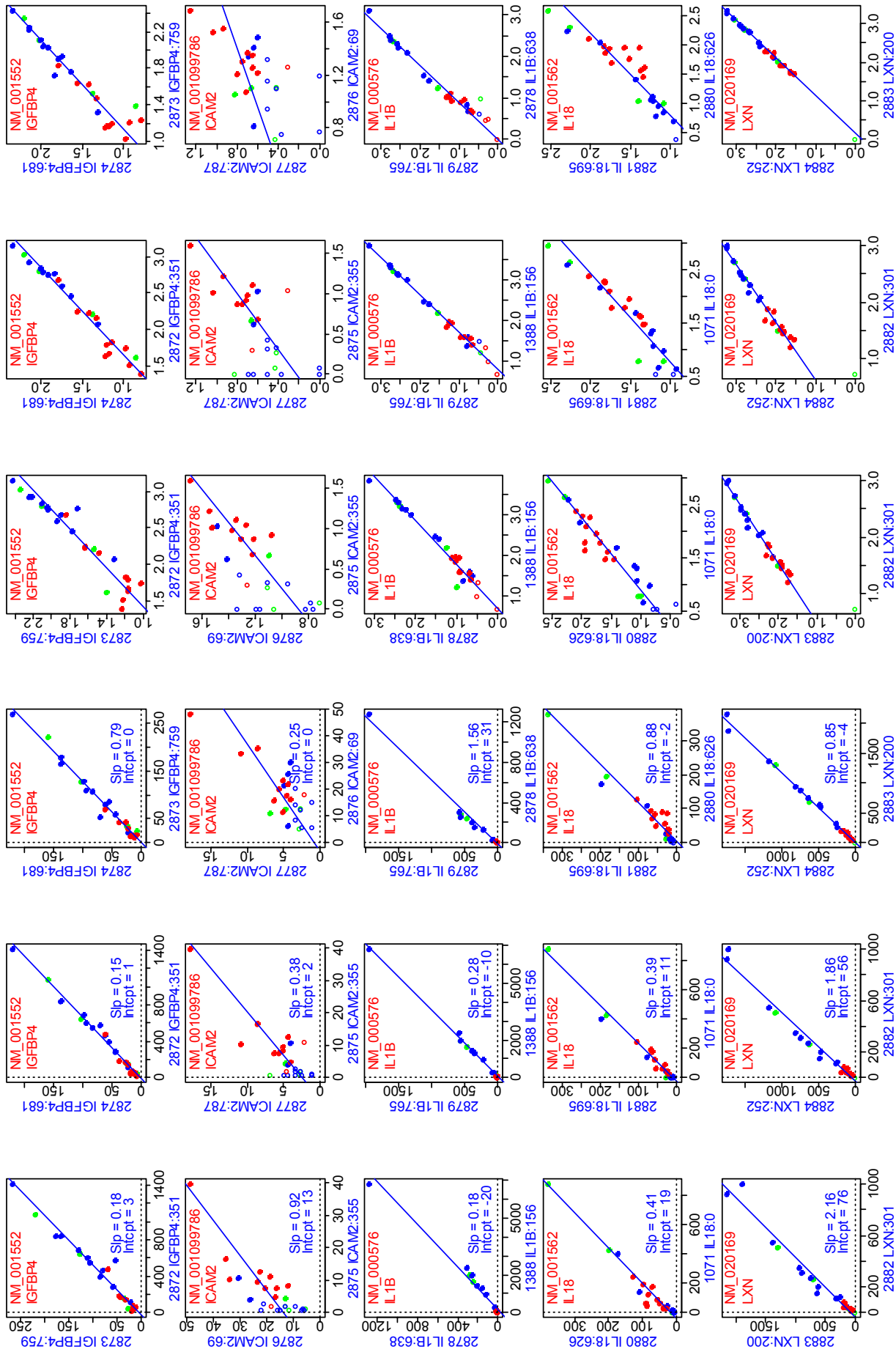

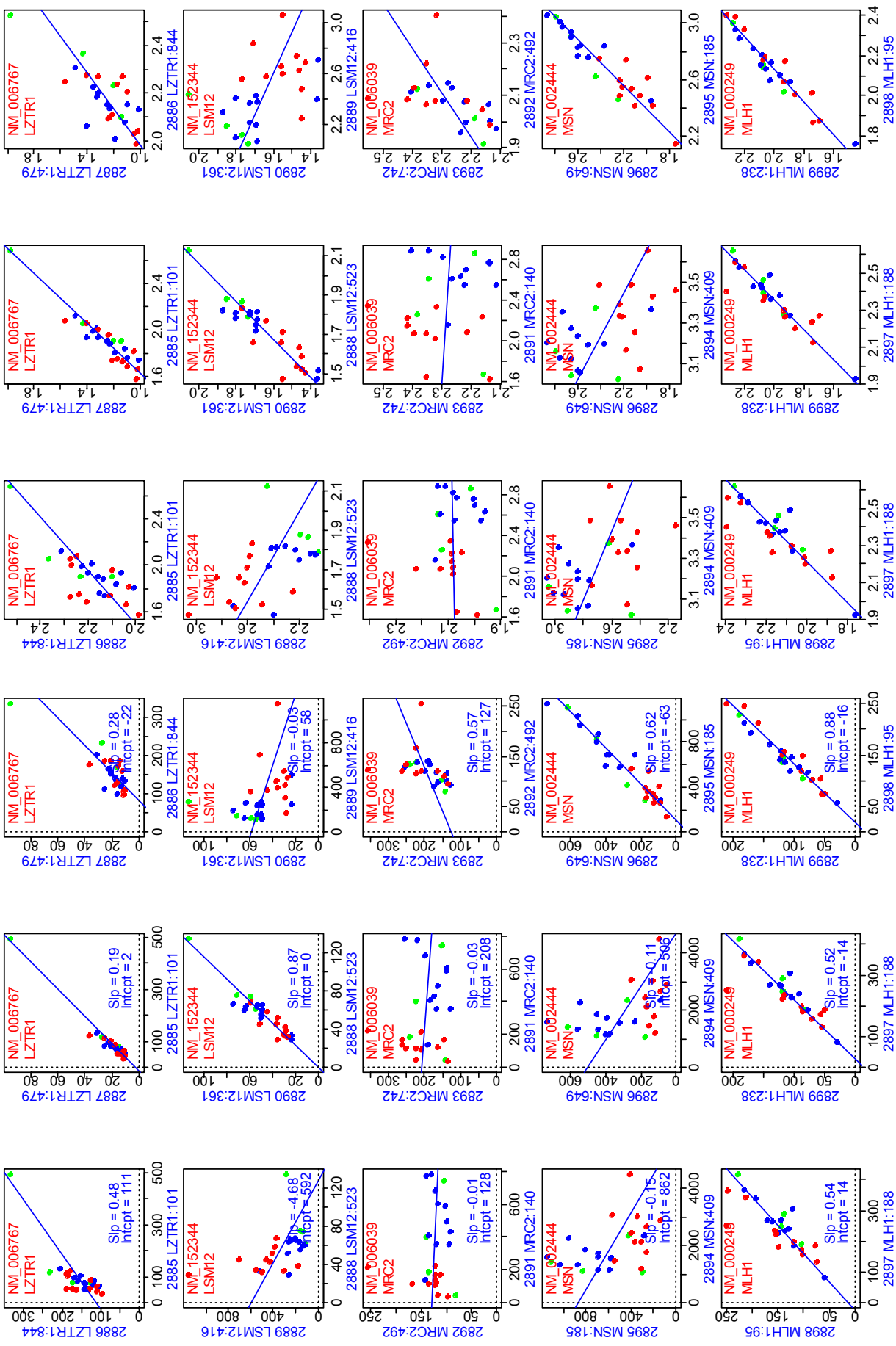

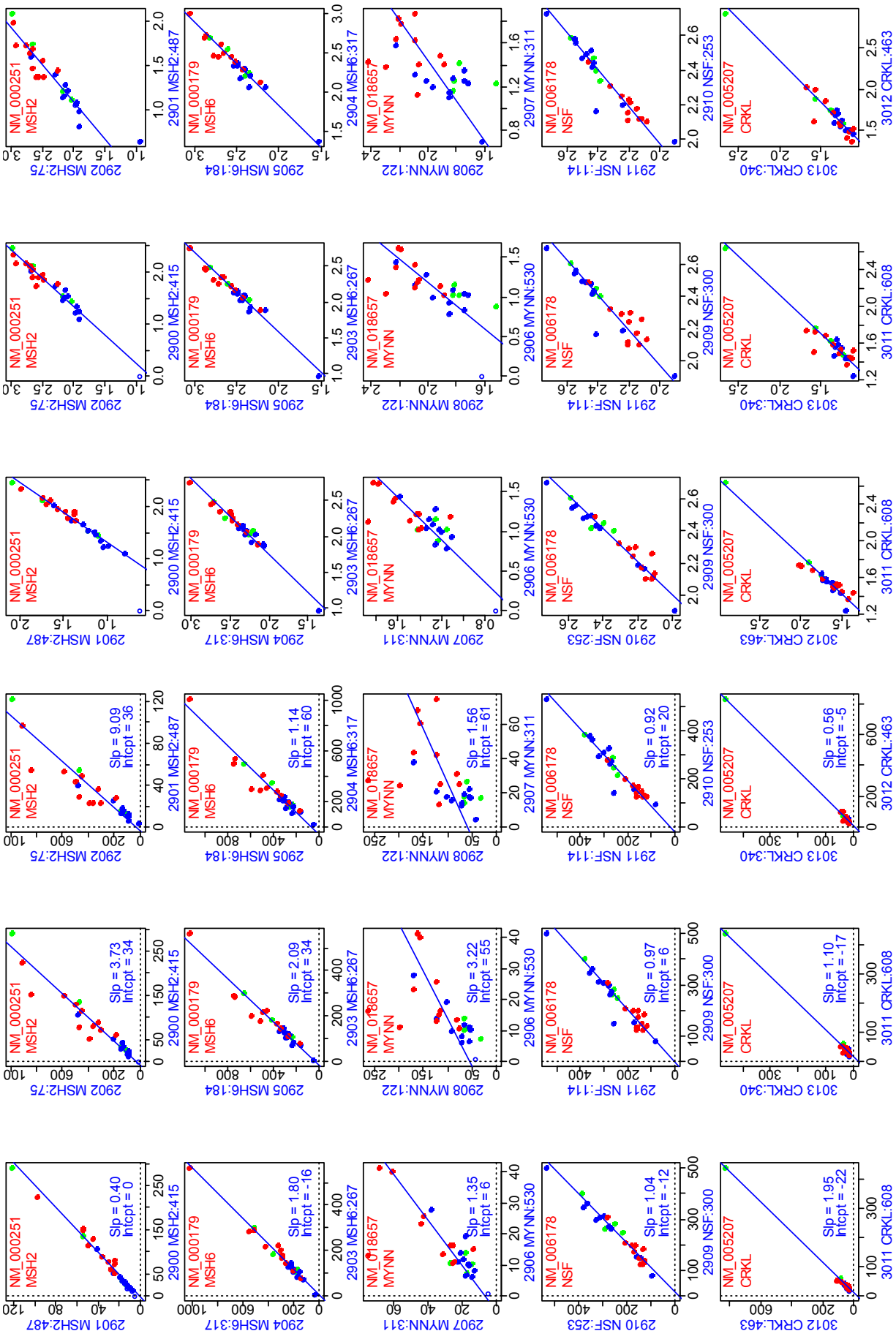

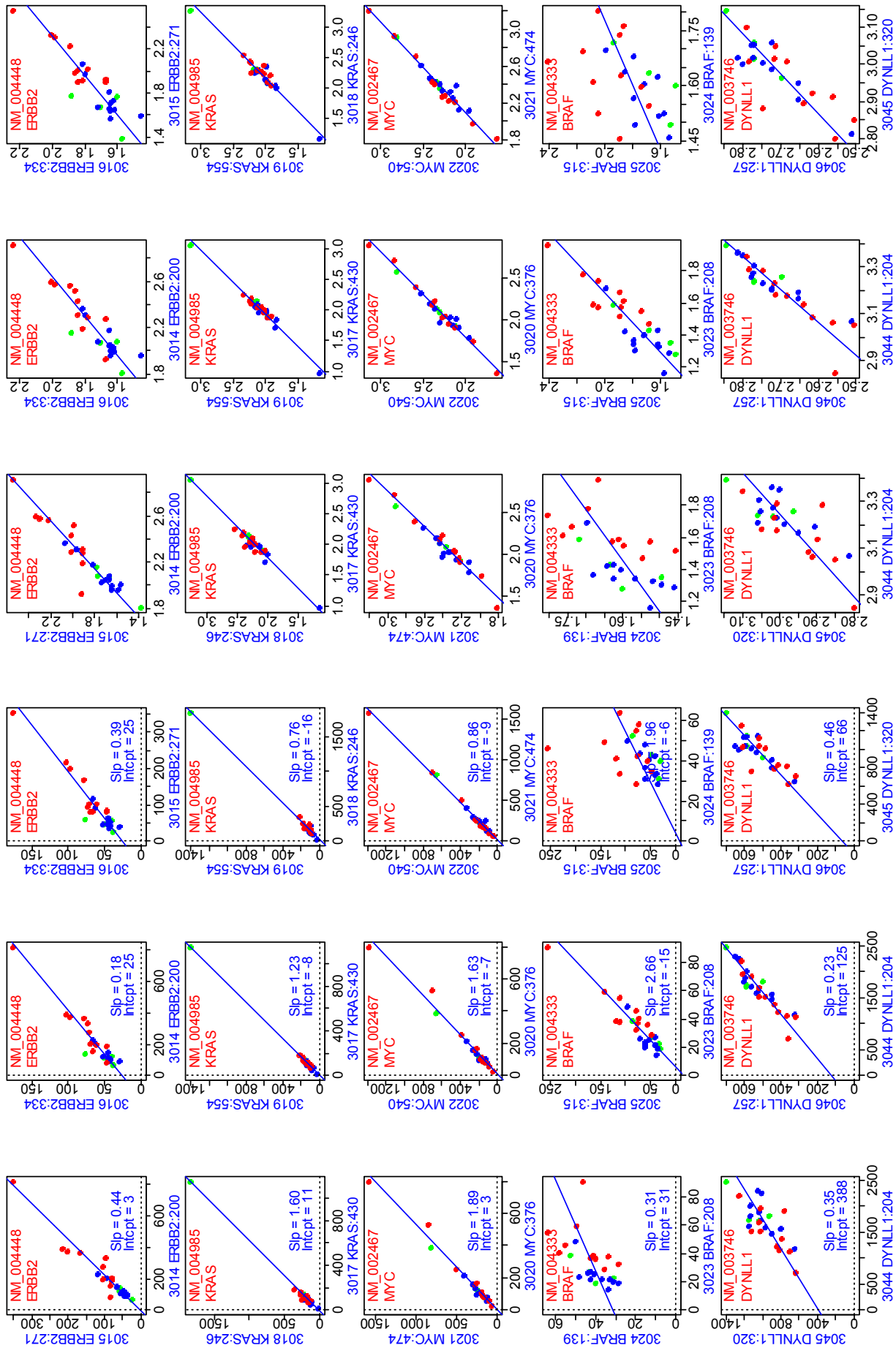

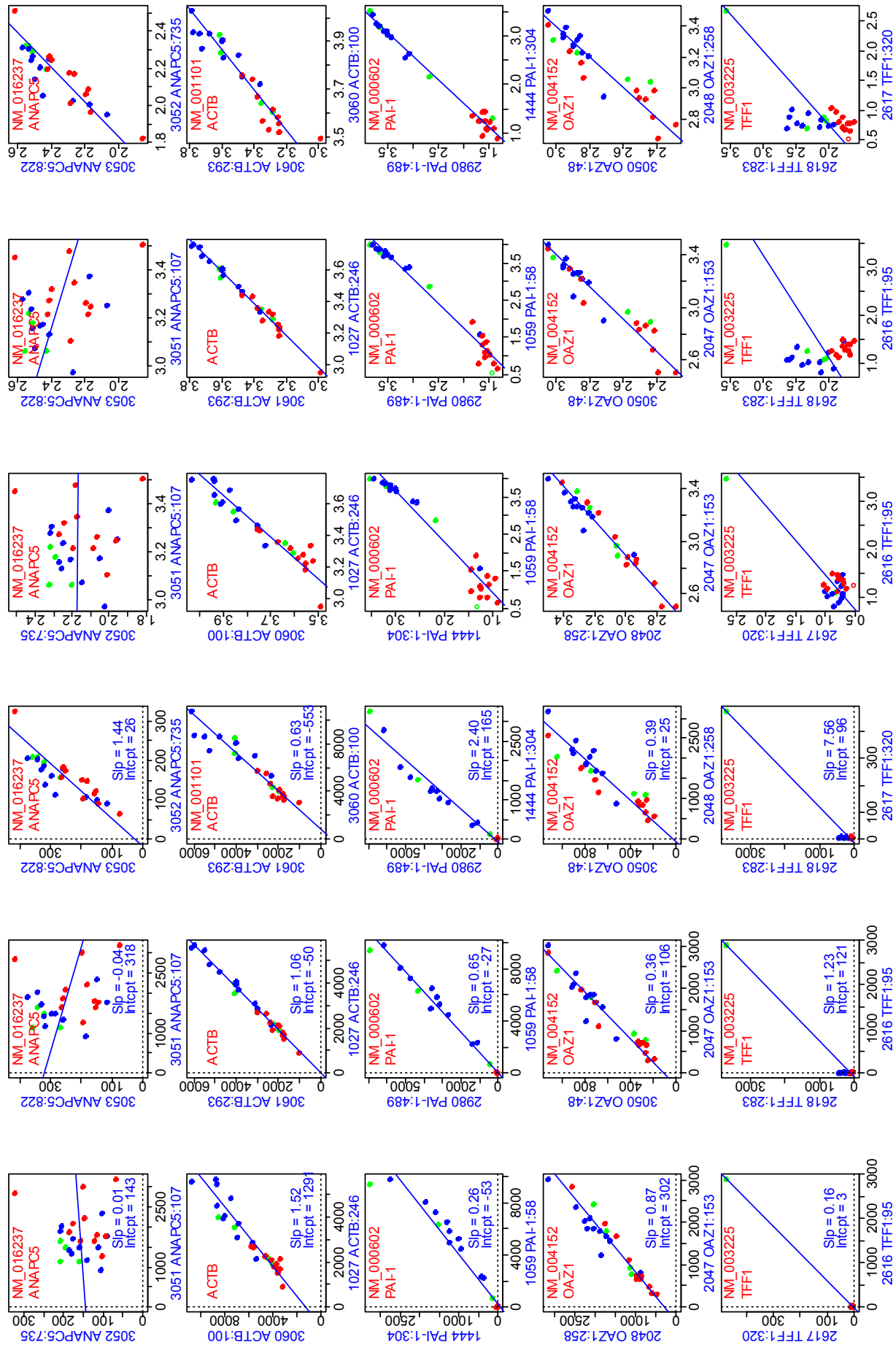

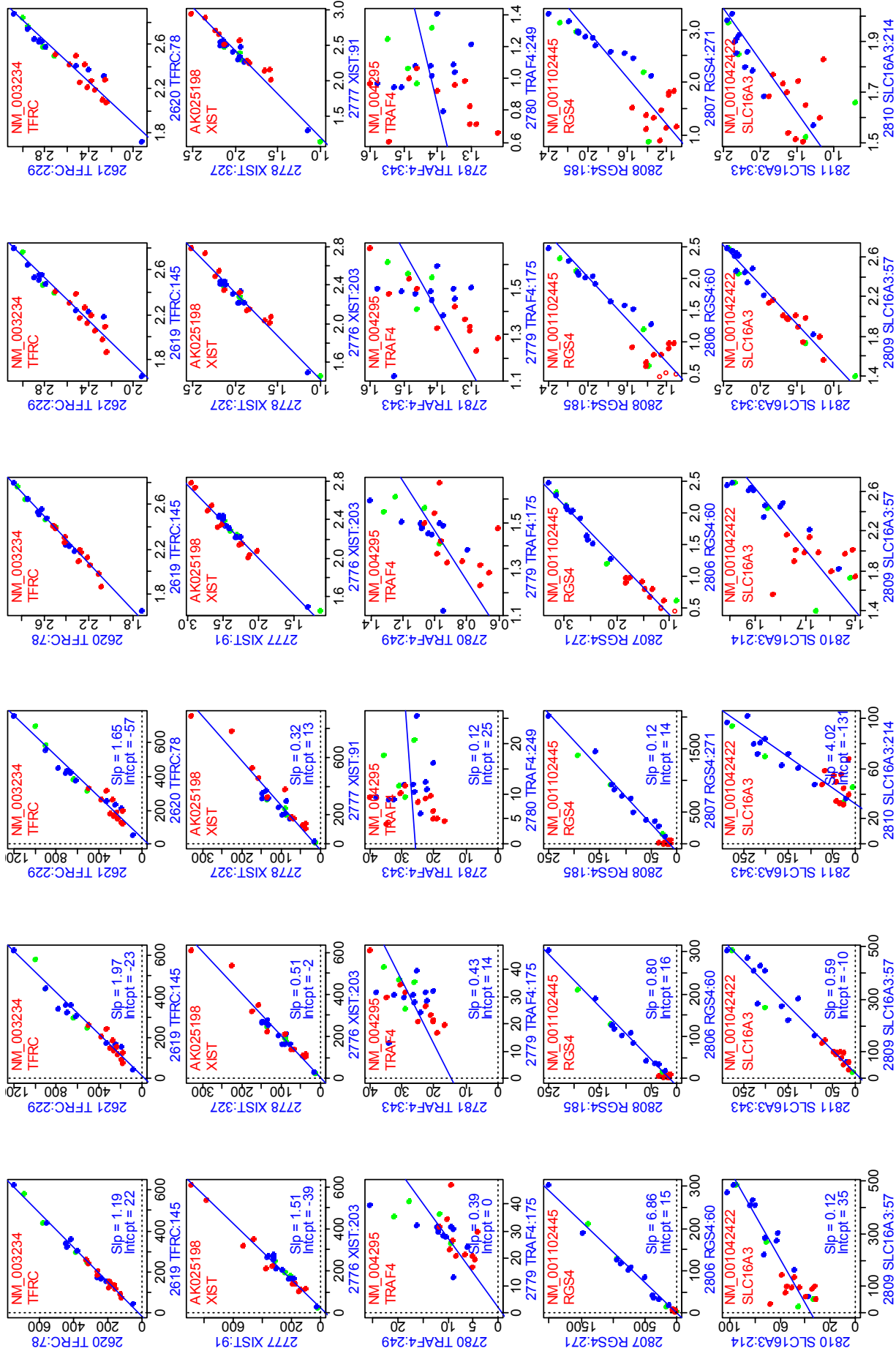

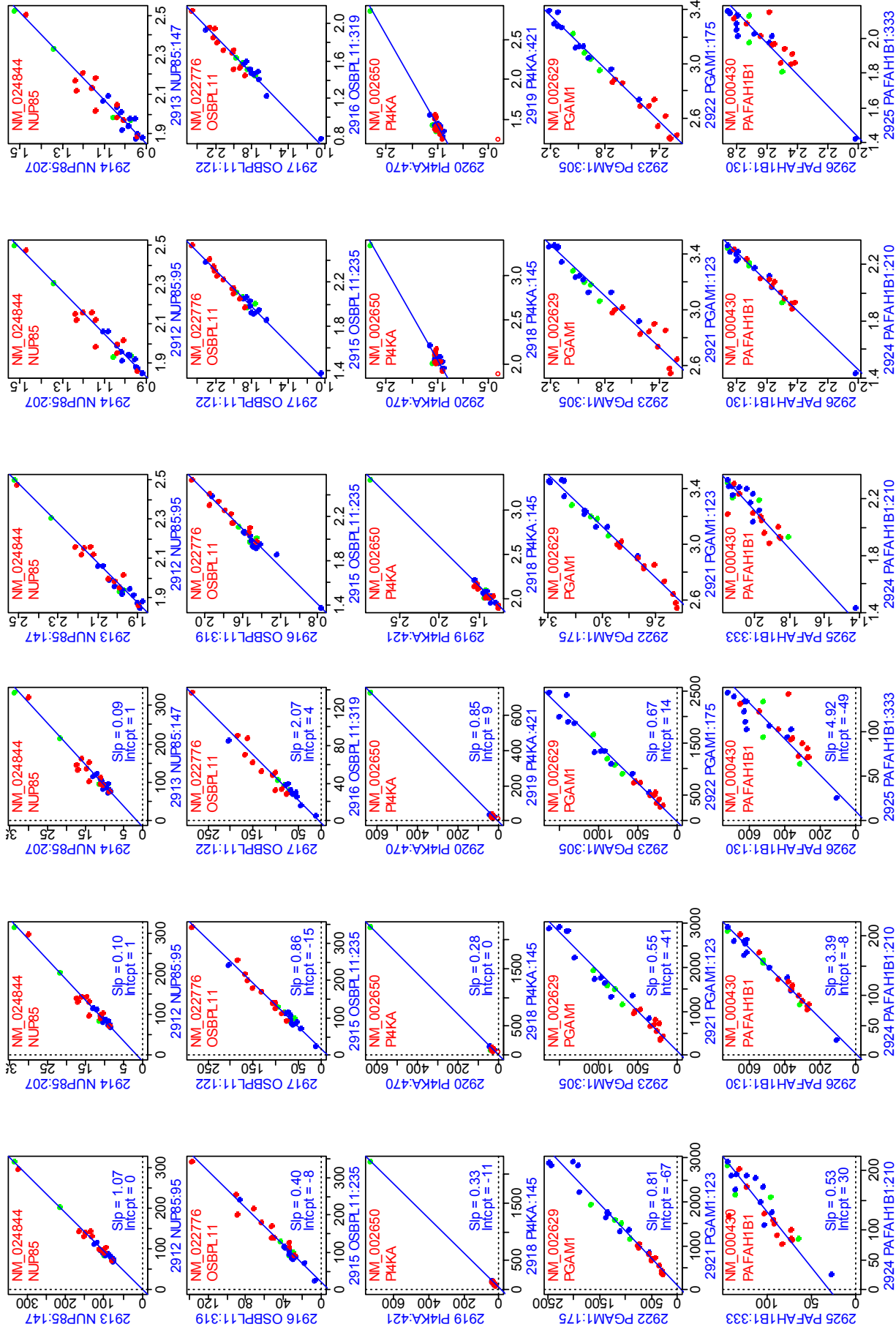

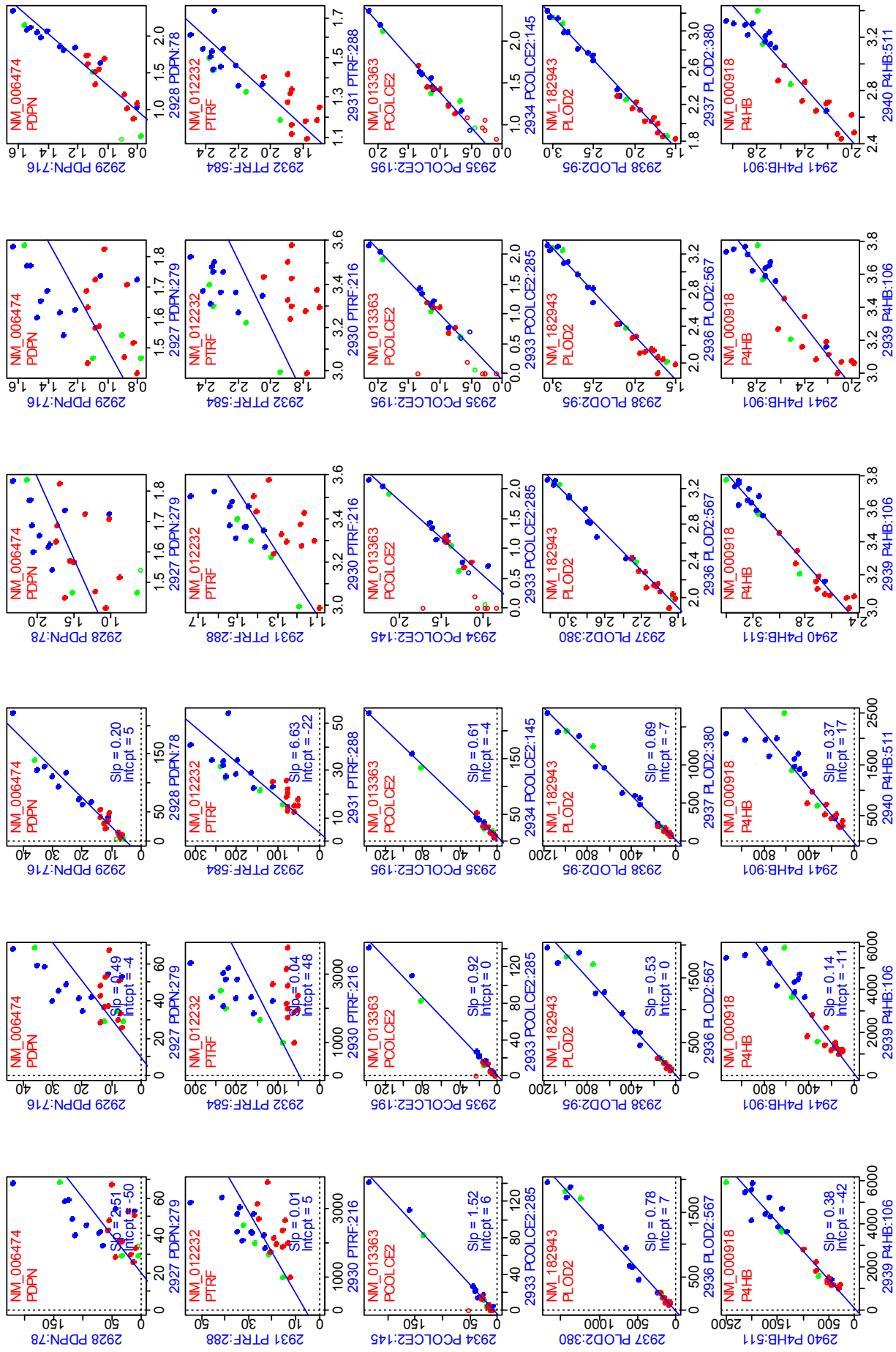

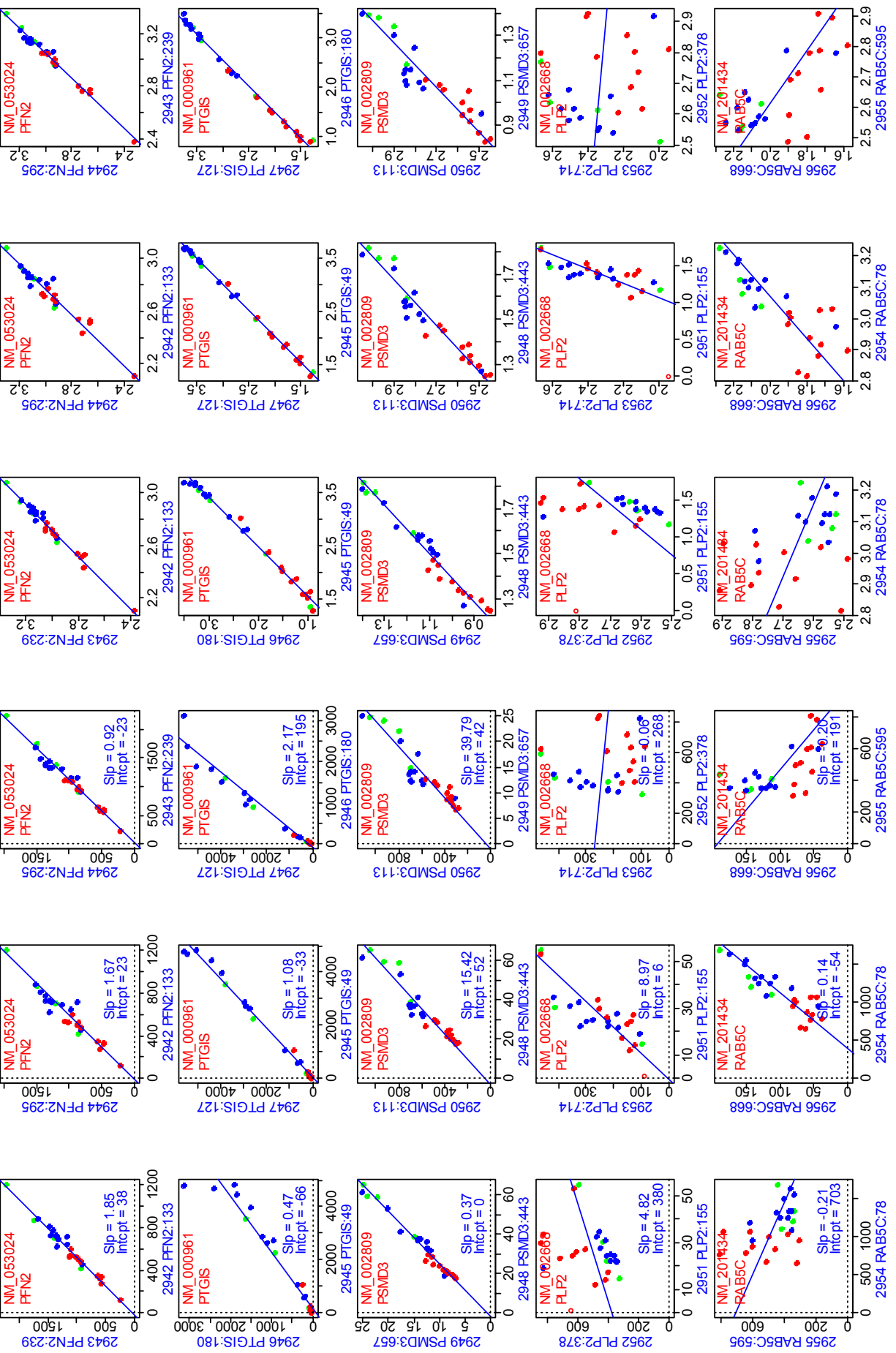

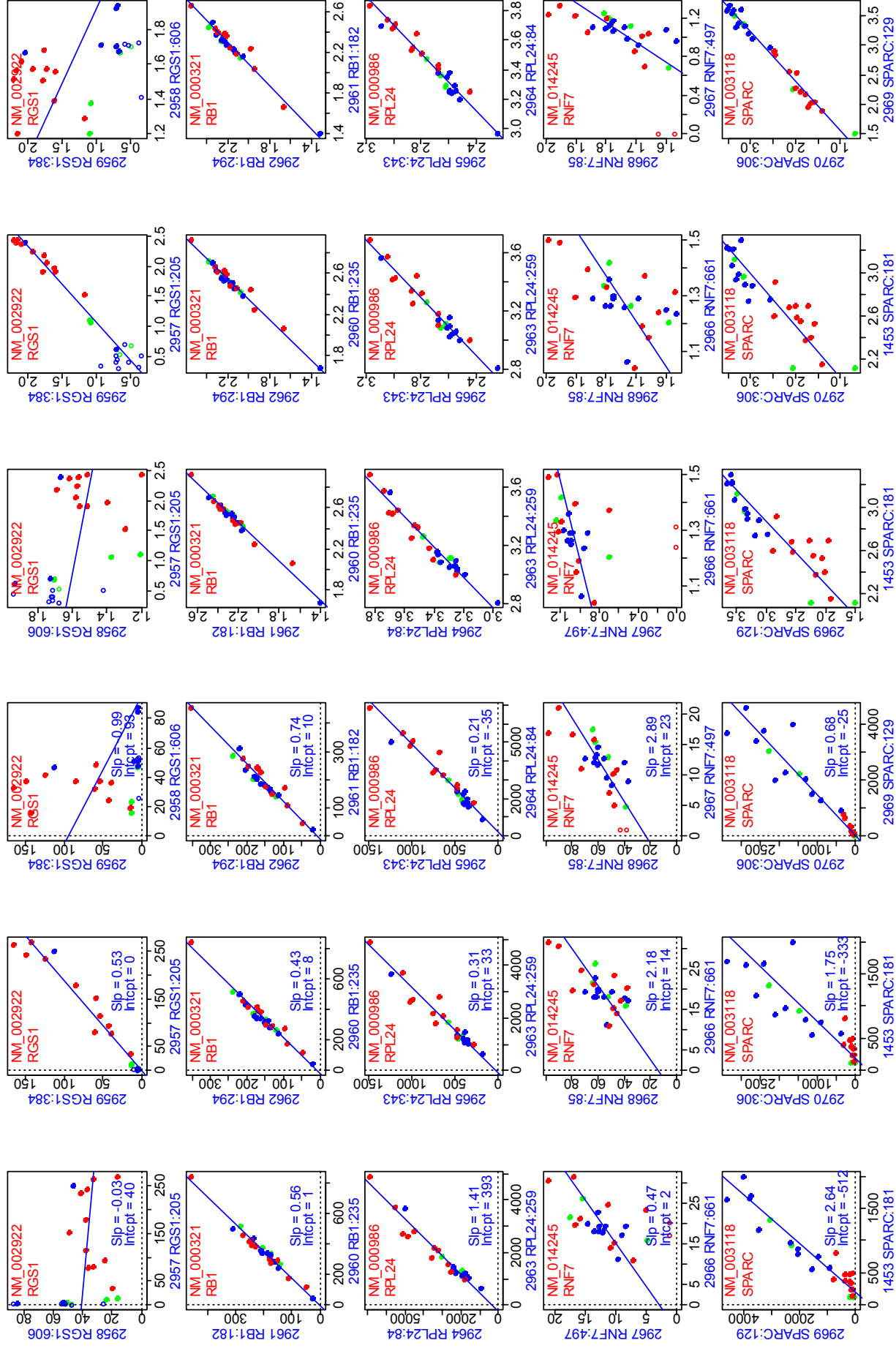

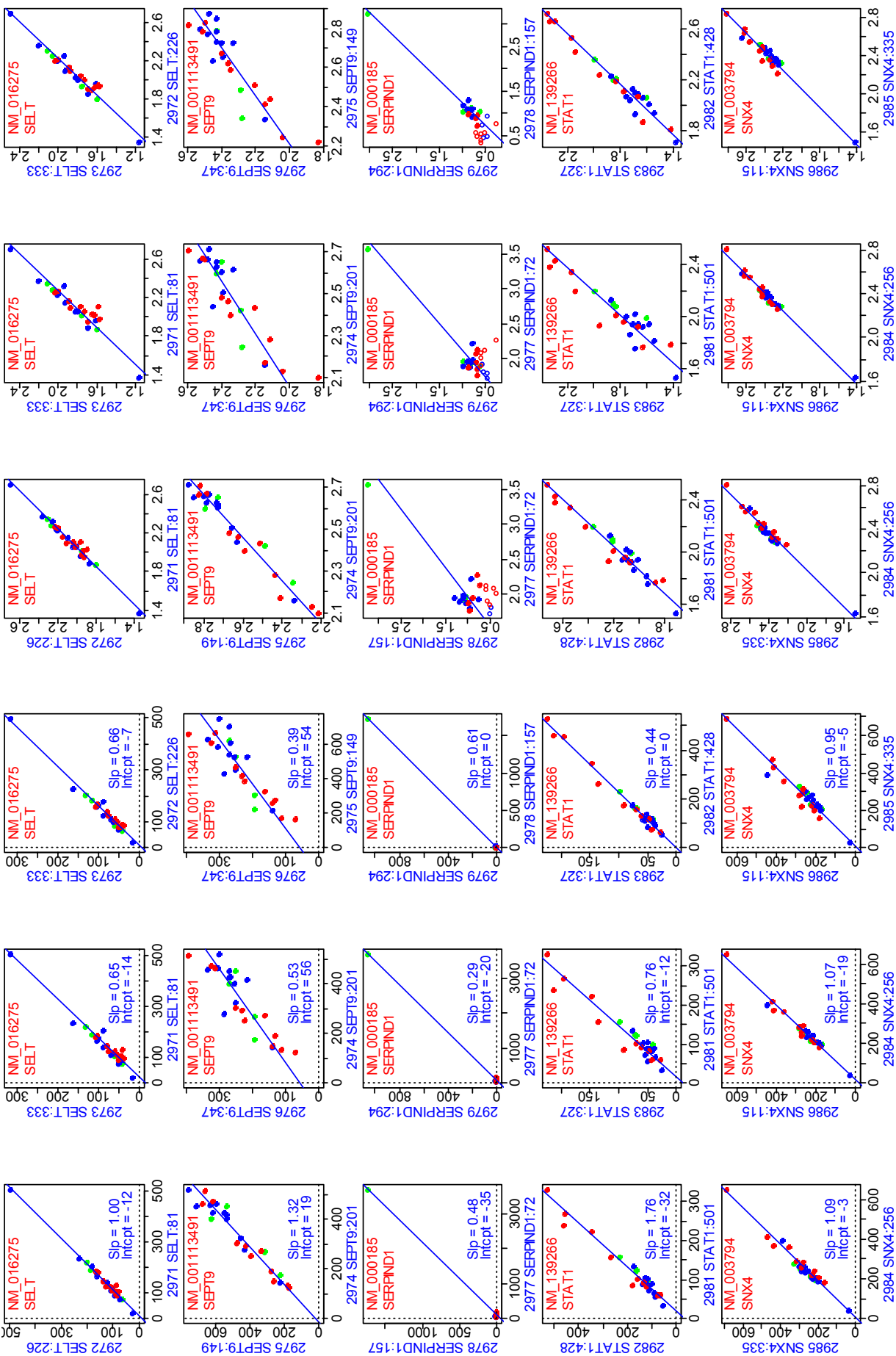

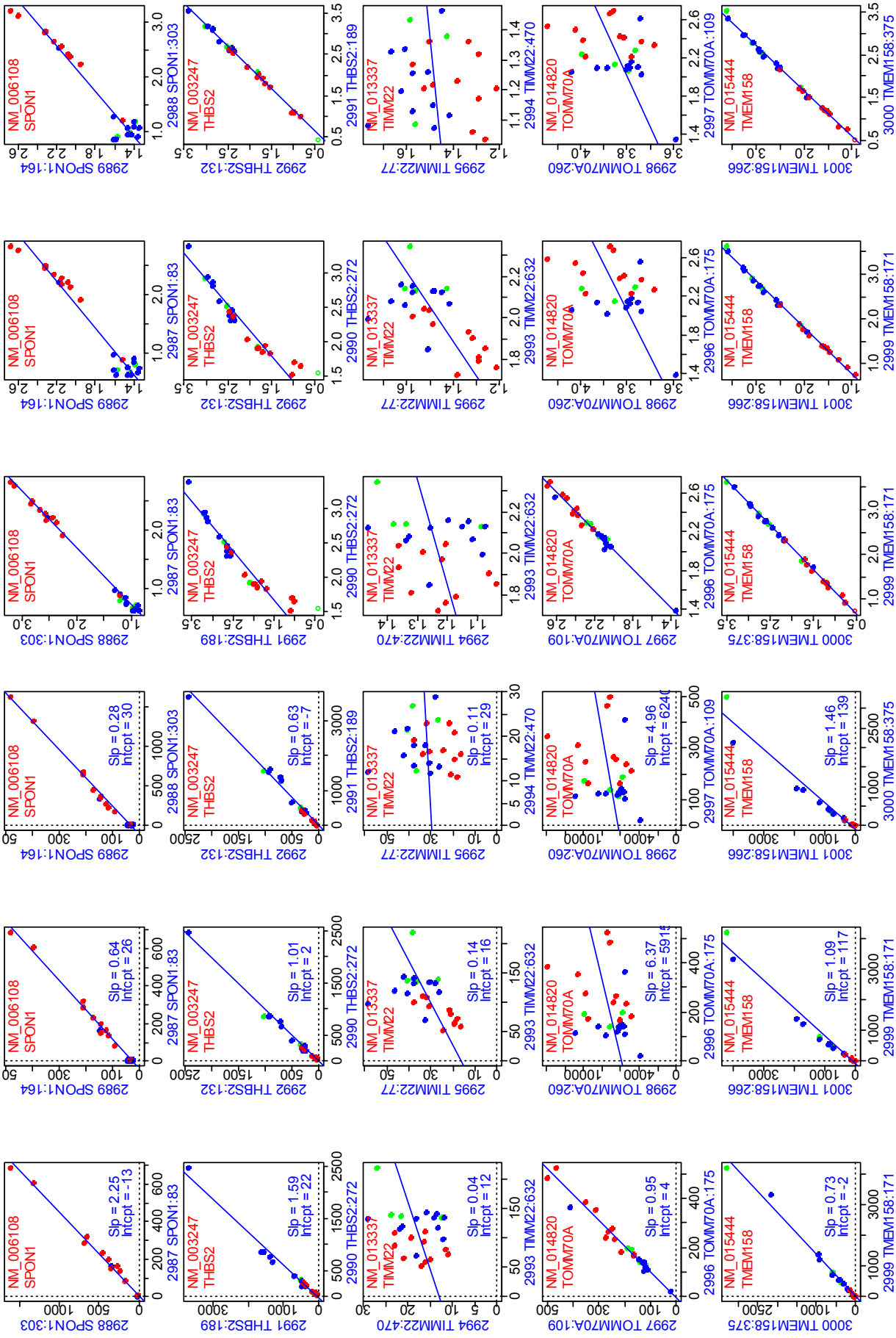

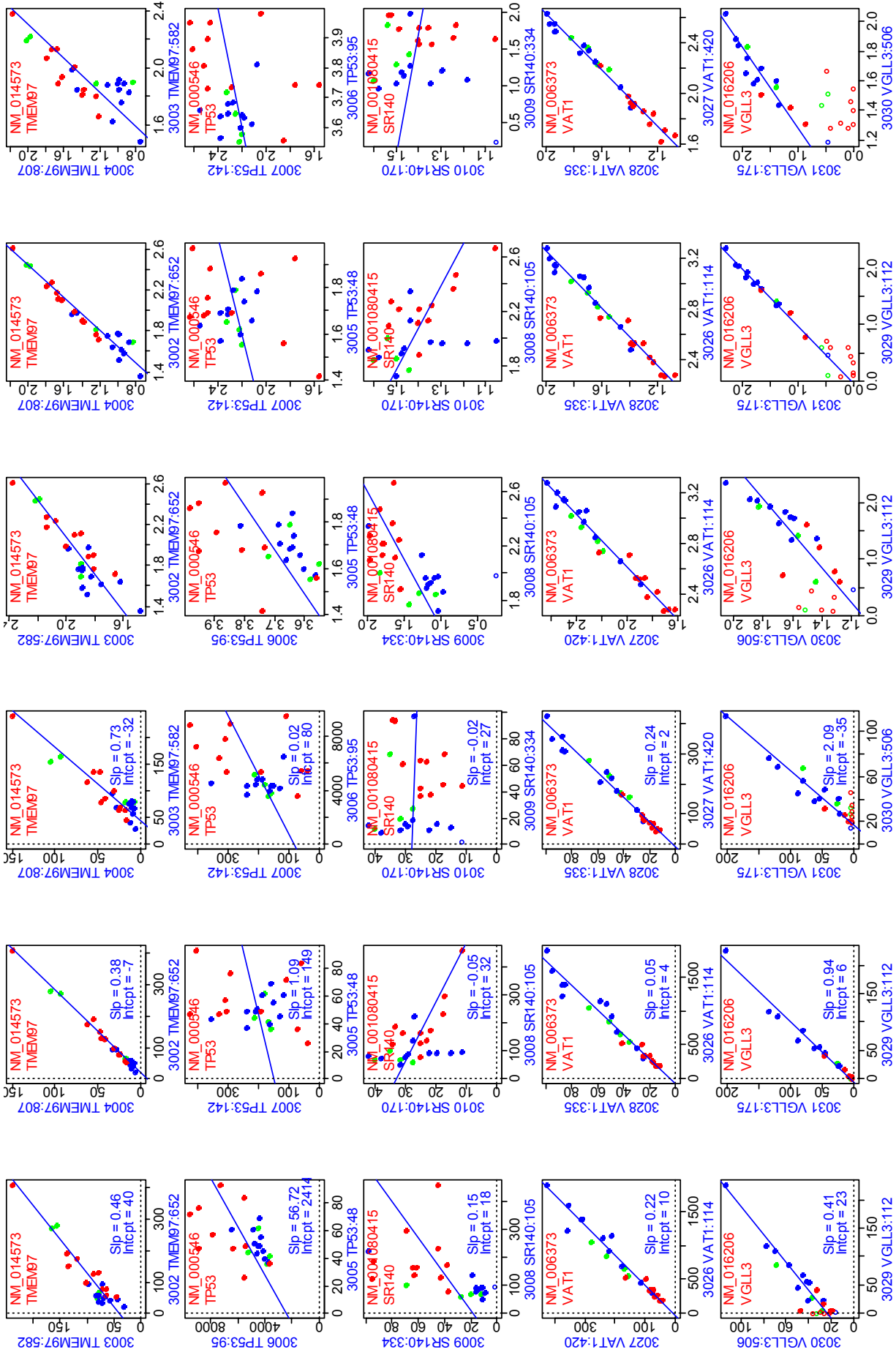

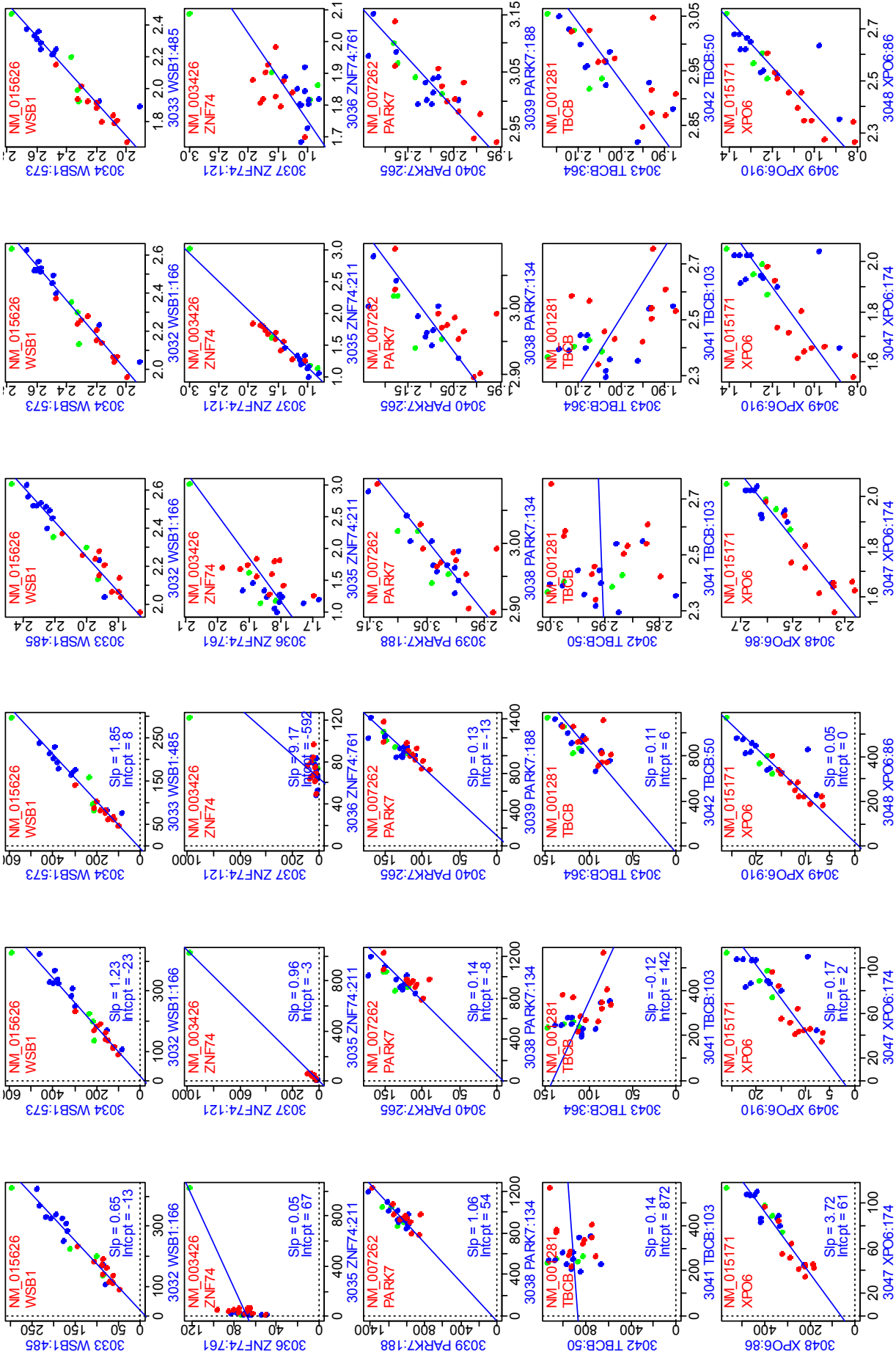

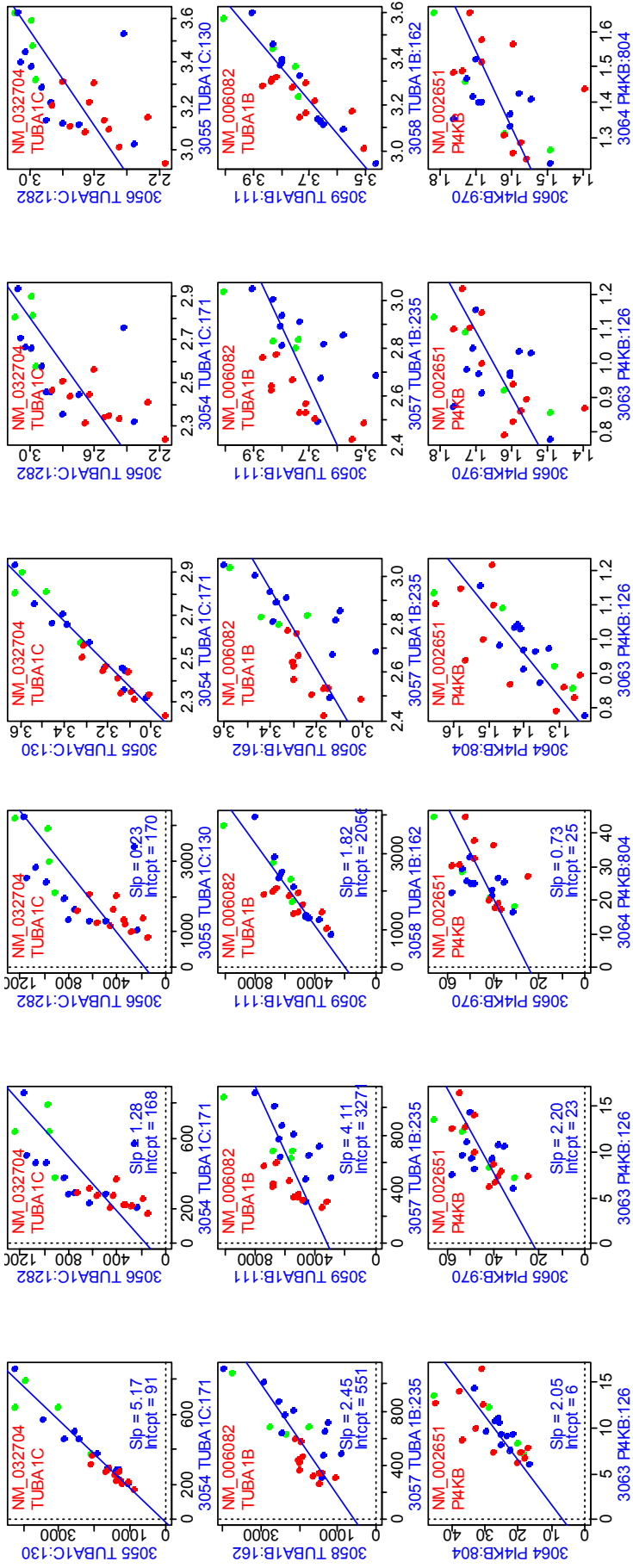

Supplement: Additional file 3 — Correlations between different probe designs for the same target gene. Three different probes were designed and tested for each of the target genes, except for one of the genes (APOE) for which there were two designs. Each row of plots contains correlations between probes for a given gene. The accession numbers and gene symbols are indicated on the plots. Plots with linear scales are shown on the left, and plots with log10scales are shown on the right. The probes are identified in the axis labels with an Xceed part number and the gene symbol. The distance of each probe from the 3' end of the sequence corresponding to the accession number is shown after the colon in the axis labels. The colors used to plot the data for each sample are: NOSE samples – blue, TOV samples – red, cell line samples – green. Low intensity probes are plotted with open symbols. [file 1479-5876-7-55-S3.pdf]

## Slide 1
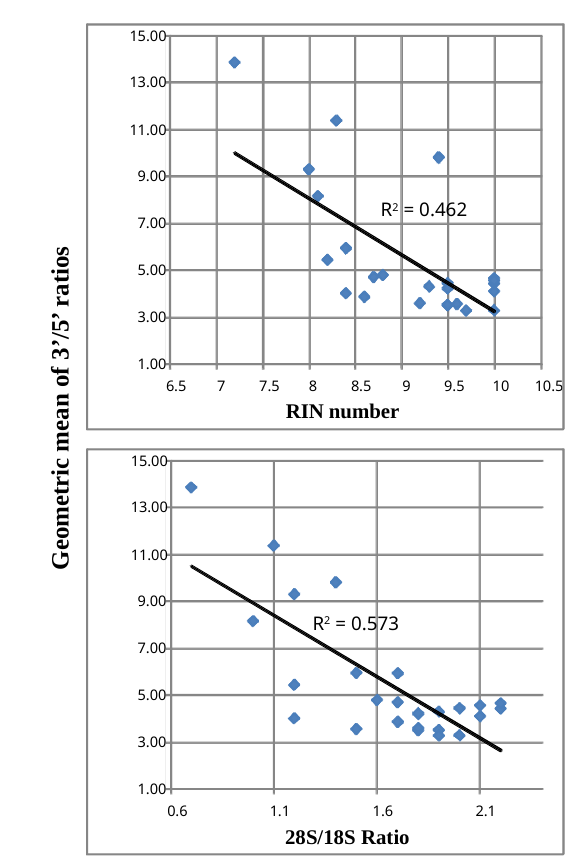

15.00
13.00
11.00
9.00
R2 = 0.462
7.00
5.00
3.00
Geometric mean of 3’/5’ ratios
1.00
6.5
7
7.5
8
8.5
9
9.5
10
10.5
RIN number
15.00
13.00
11.00
9.00
R2 = 0.573
7.00
5.00
3.00
1.00
0.6
1.1
1.6
2.1
28S/18S Ratio

Supplement: Additional file 5 — RNA quality control. Correlation between the geometric mean of seven 3'/5' control probe ratios and RIN number or 28 S/18 S ratios. Samples MG0001 (TOV-21G) and MG0026 (NOSE-1181) are not included. [file 1479-5876-7-55-S5.ppt]

## Slide 1
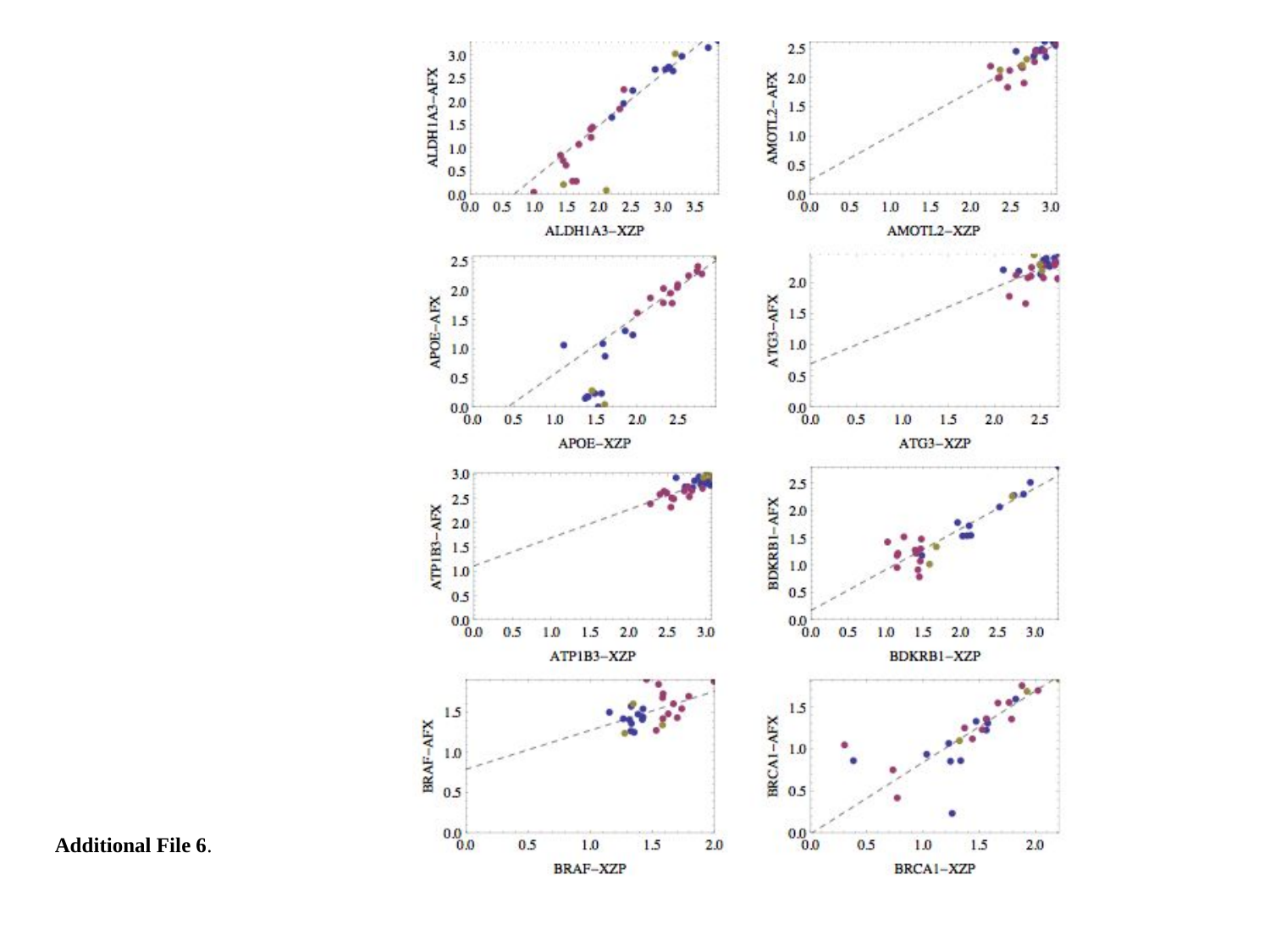

Additional File 6.

## Slide 2
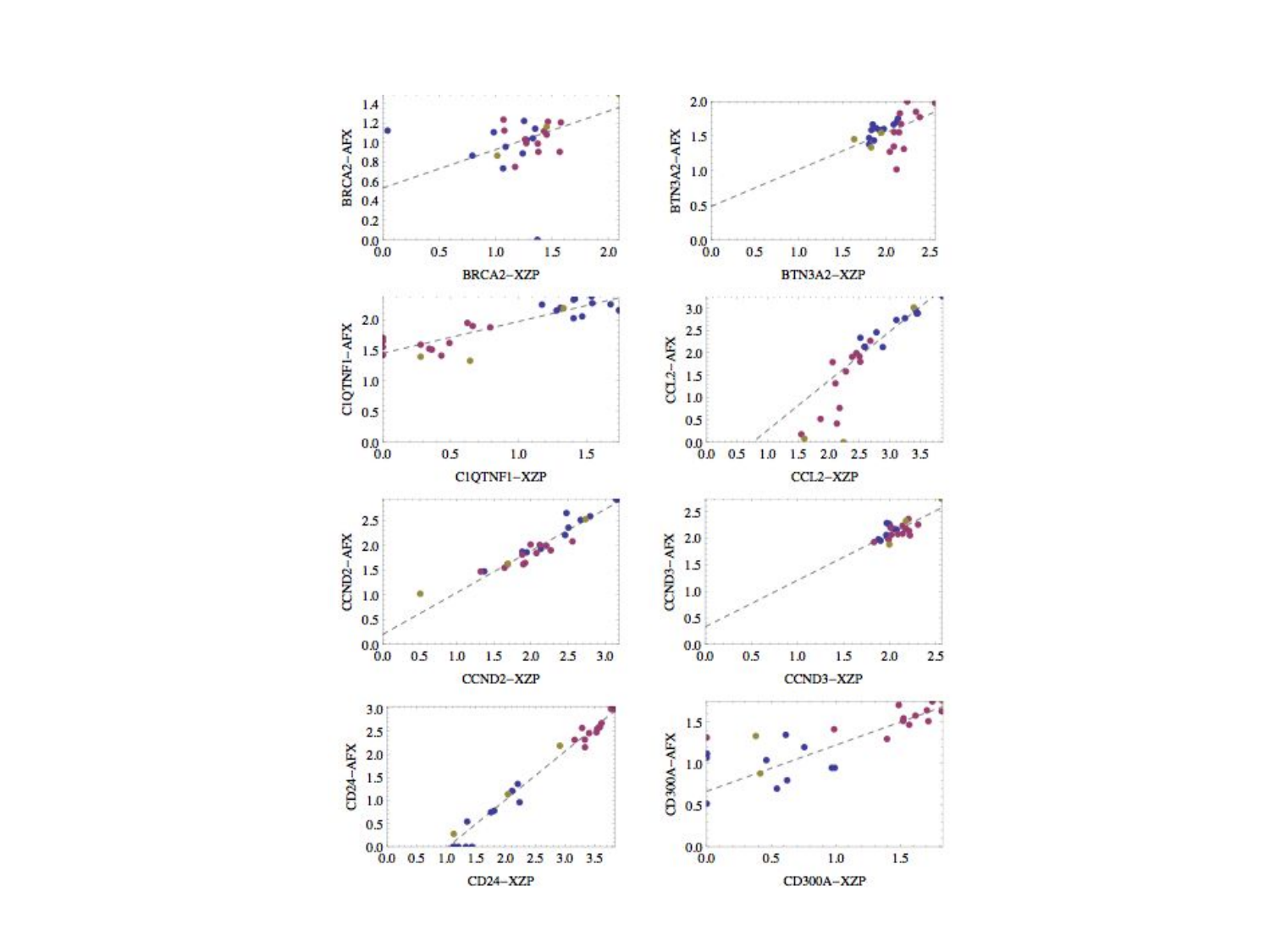

## Slide 3
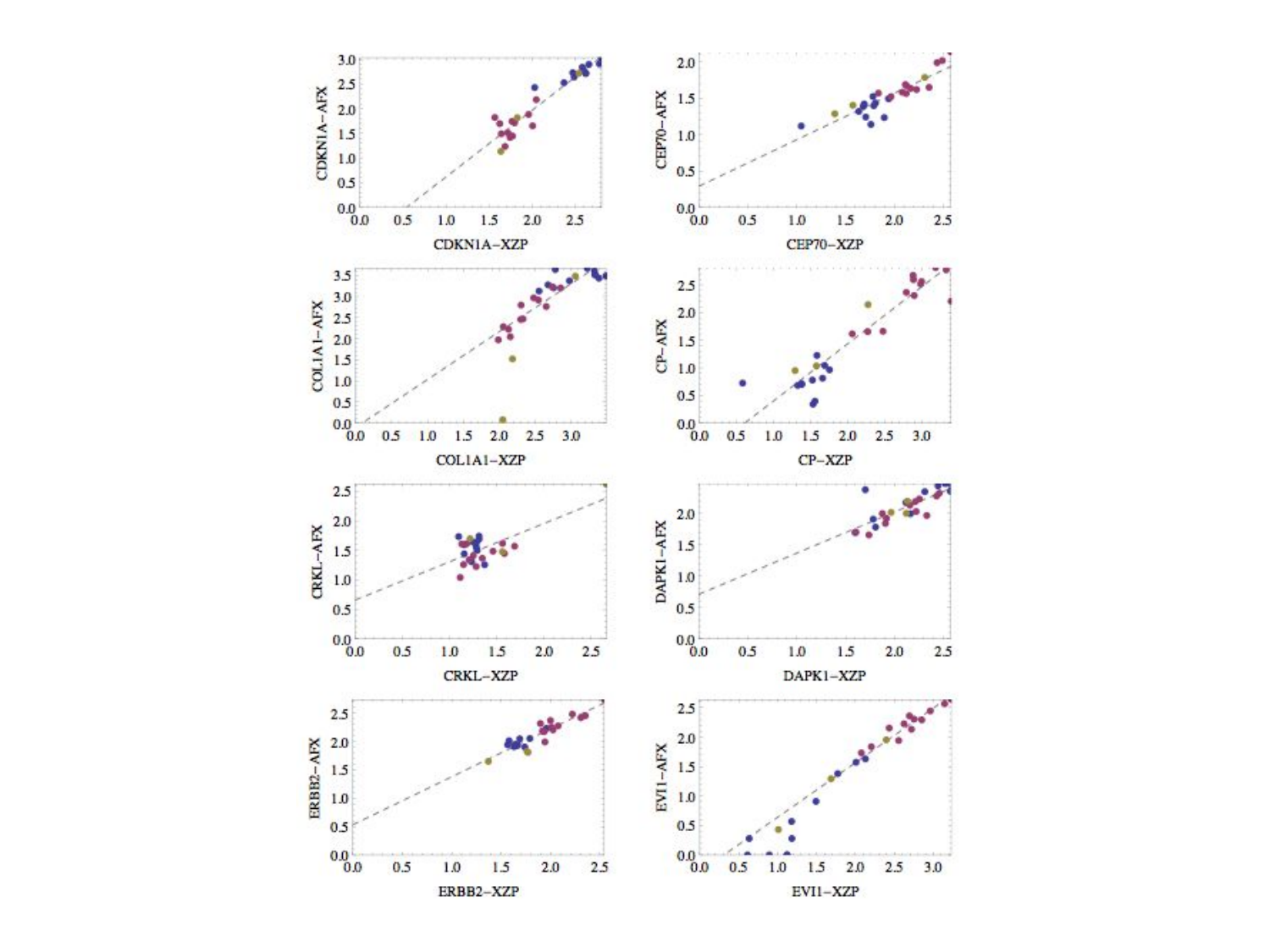

## Slide 4
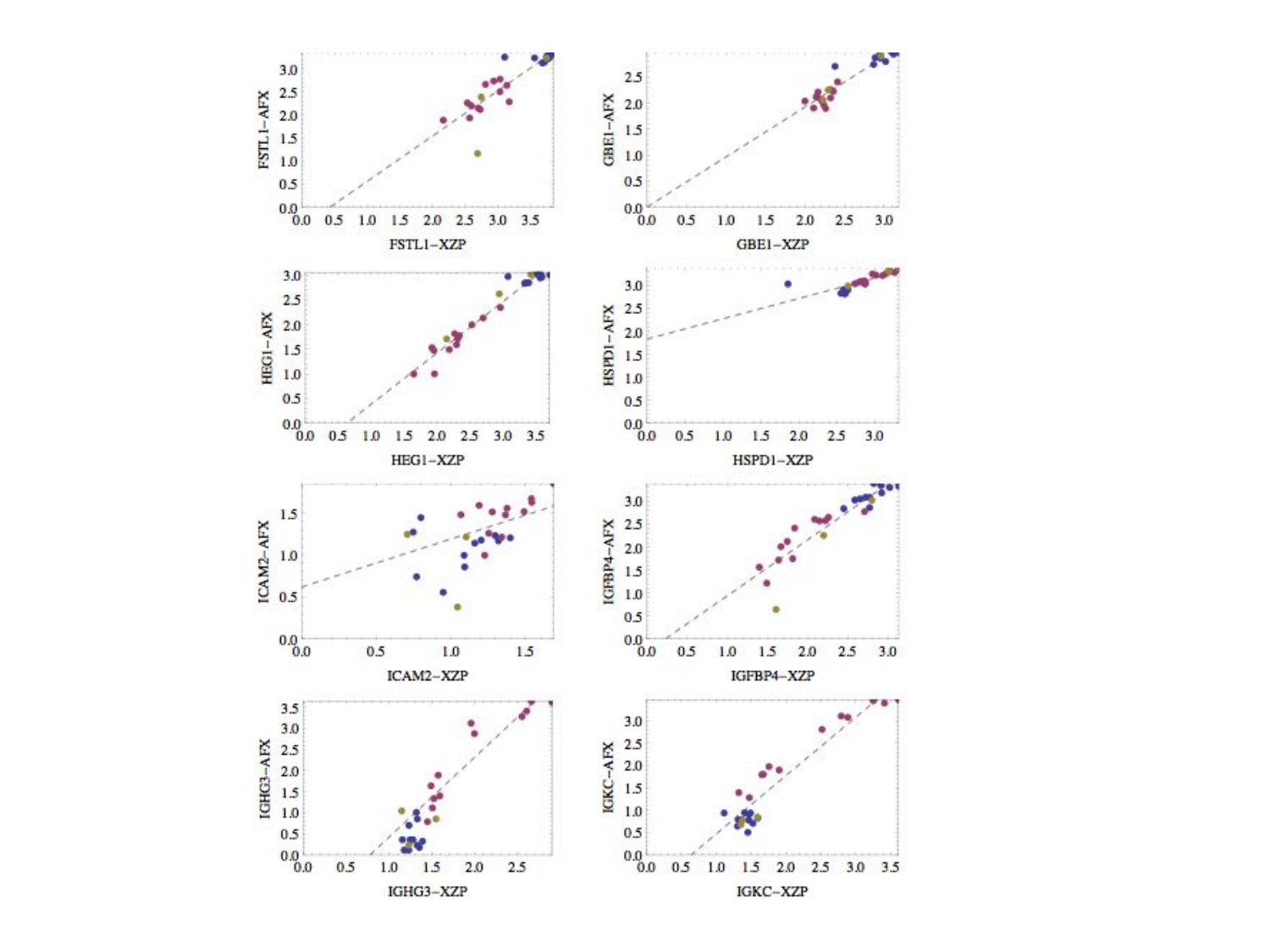

## Slide 5
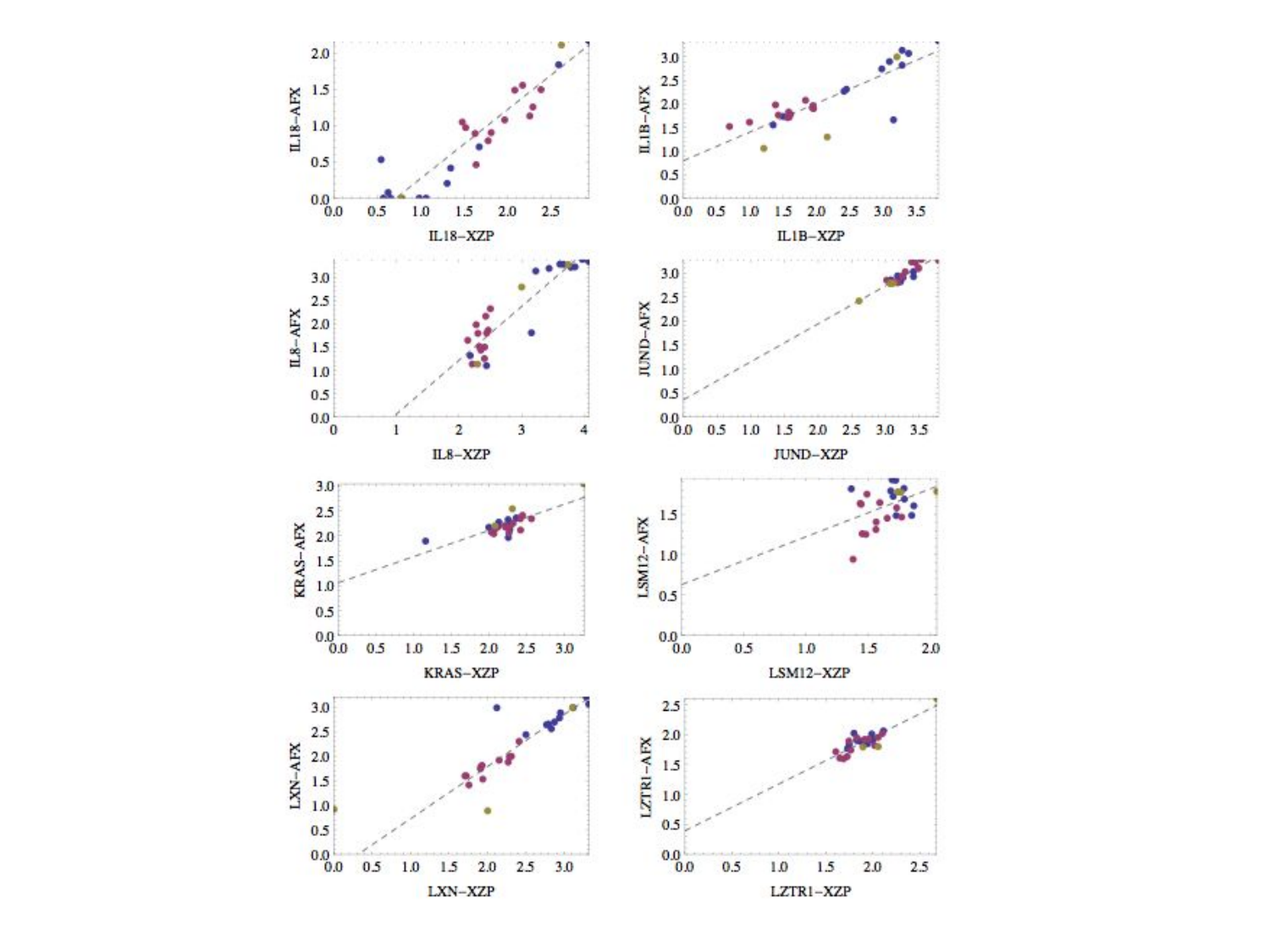

## Slide 6
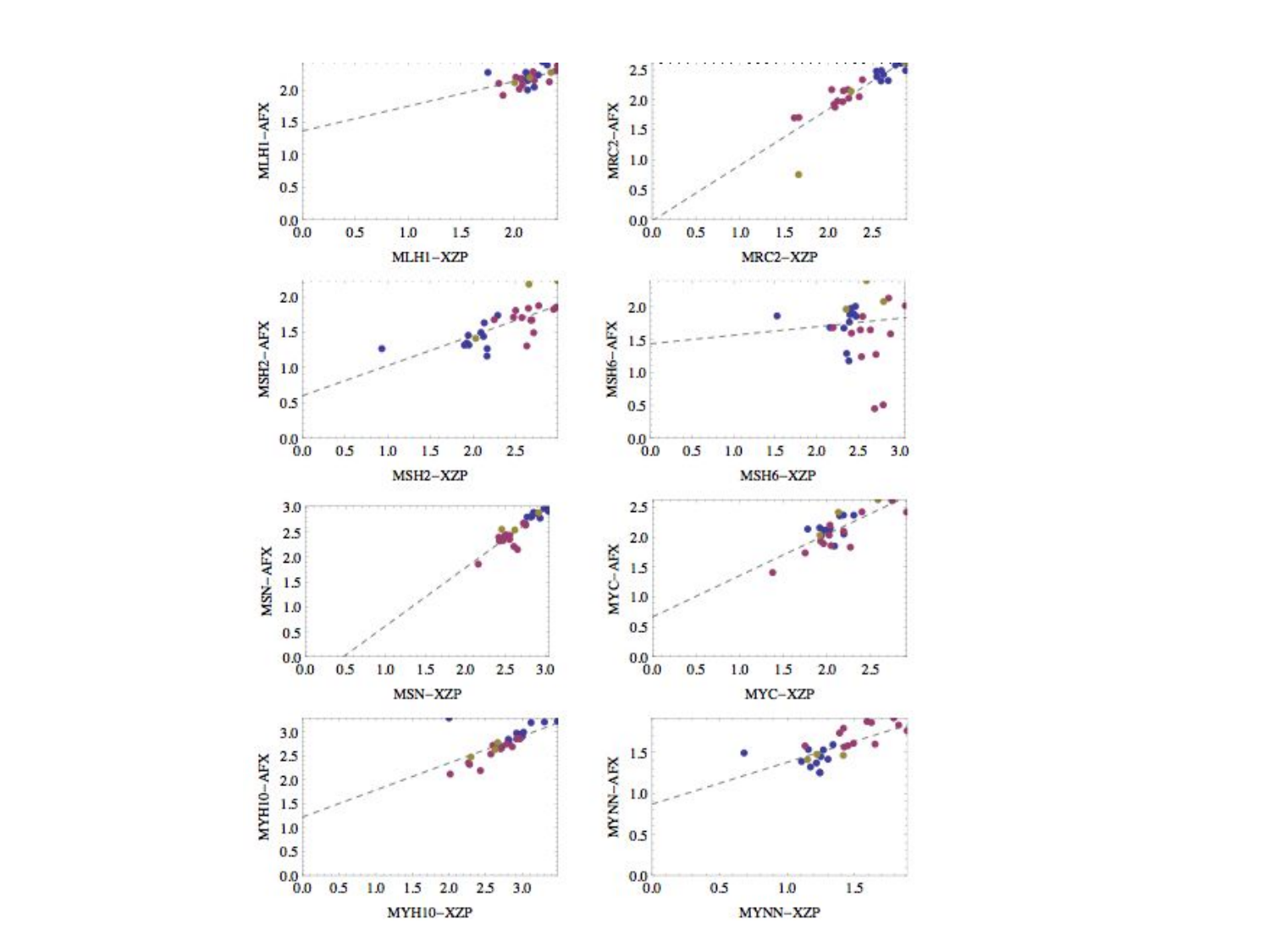

## Slide 7
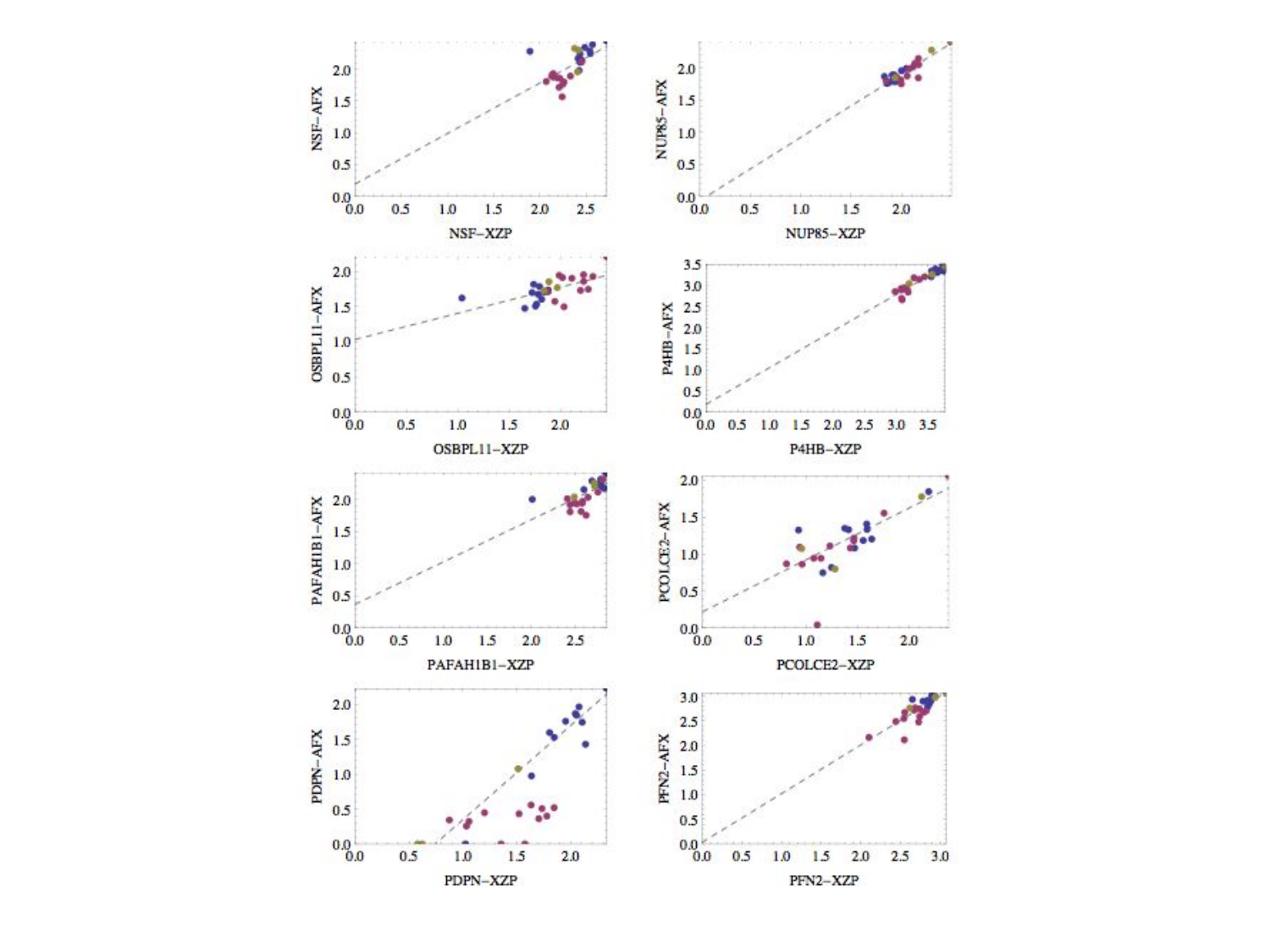

## Slide 8
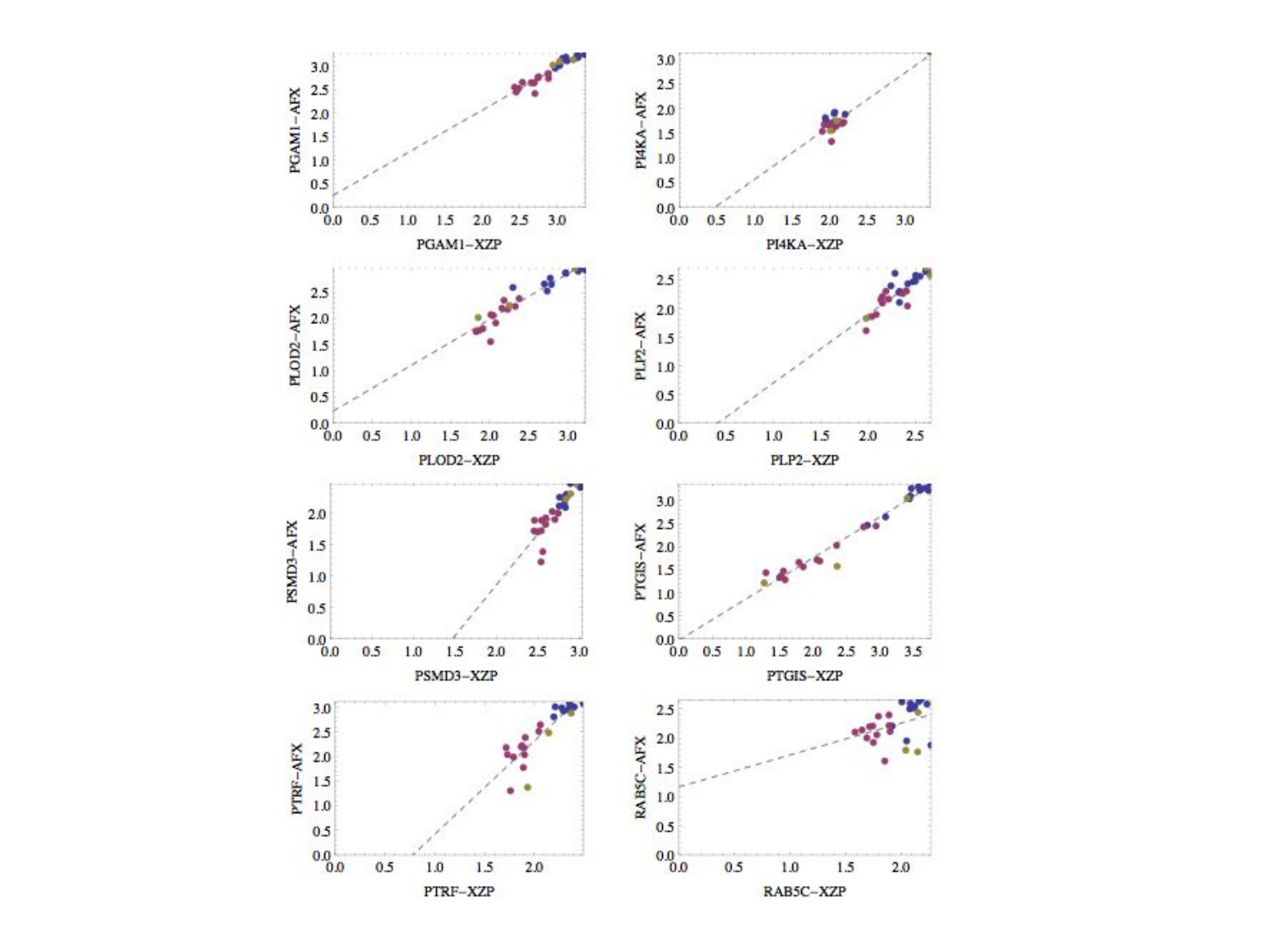

## Slide 9
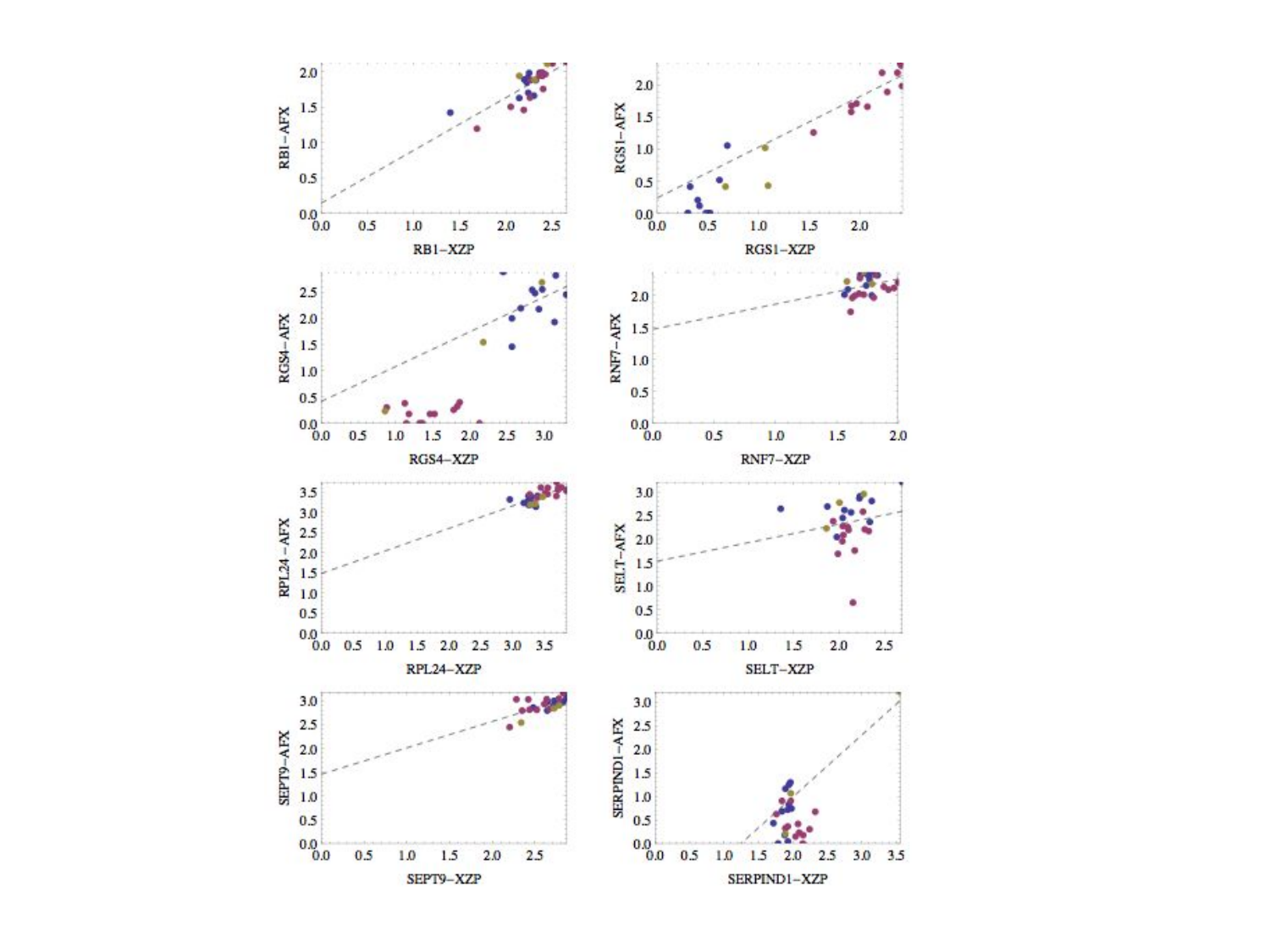

## Slide 10
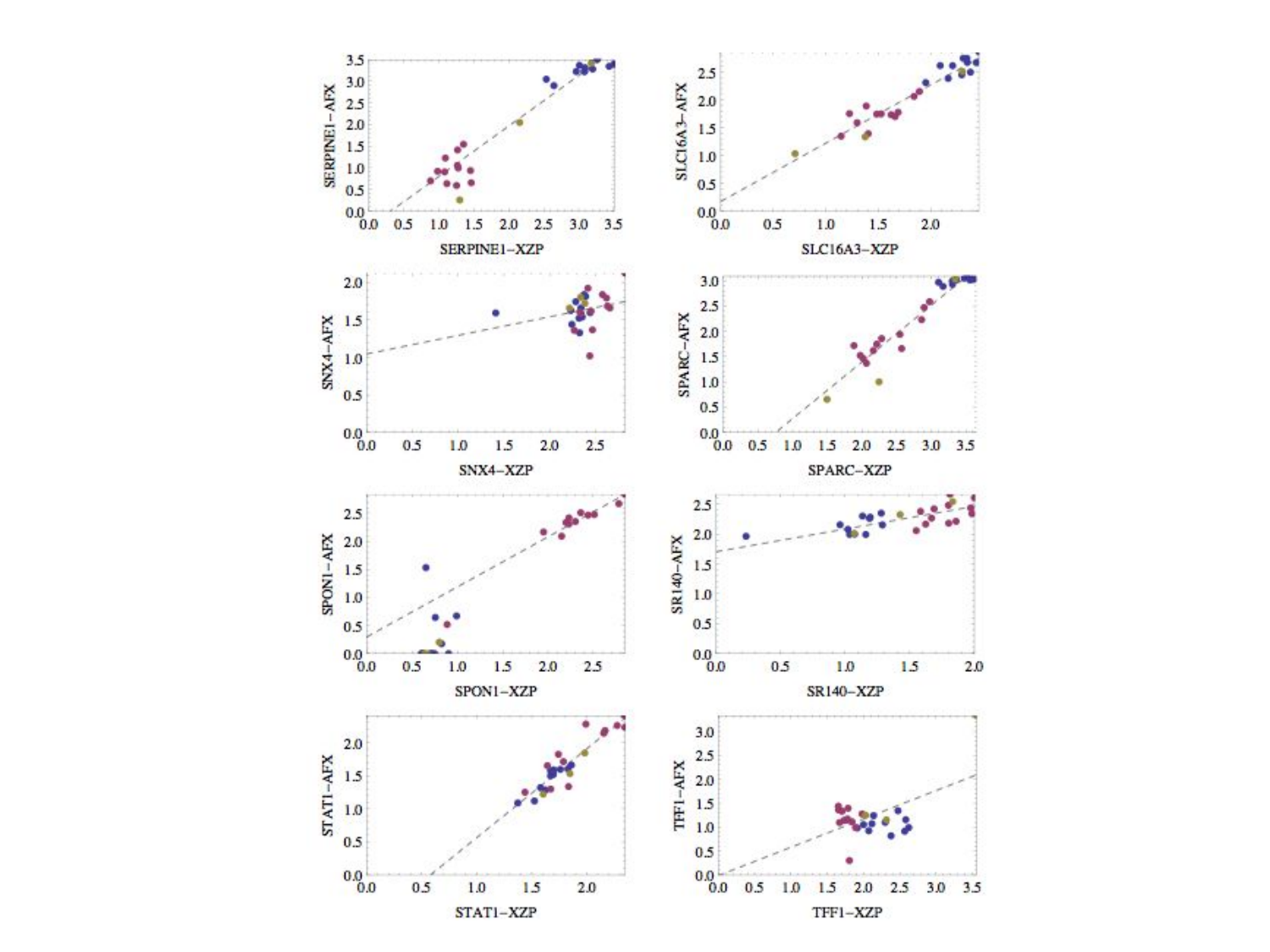

## Slide 11
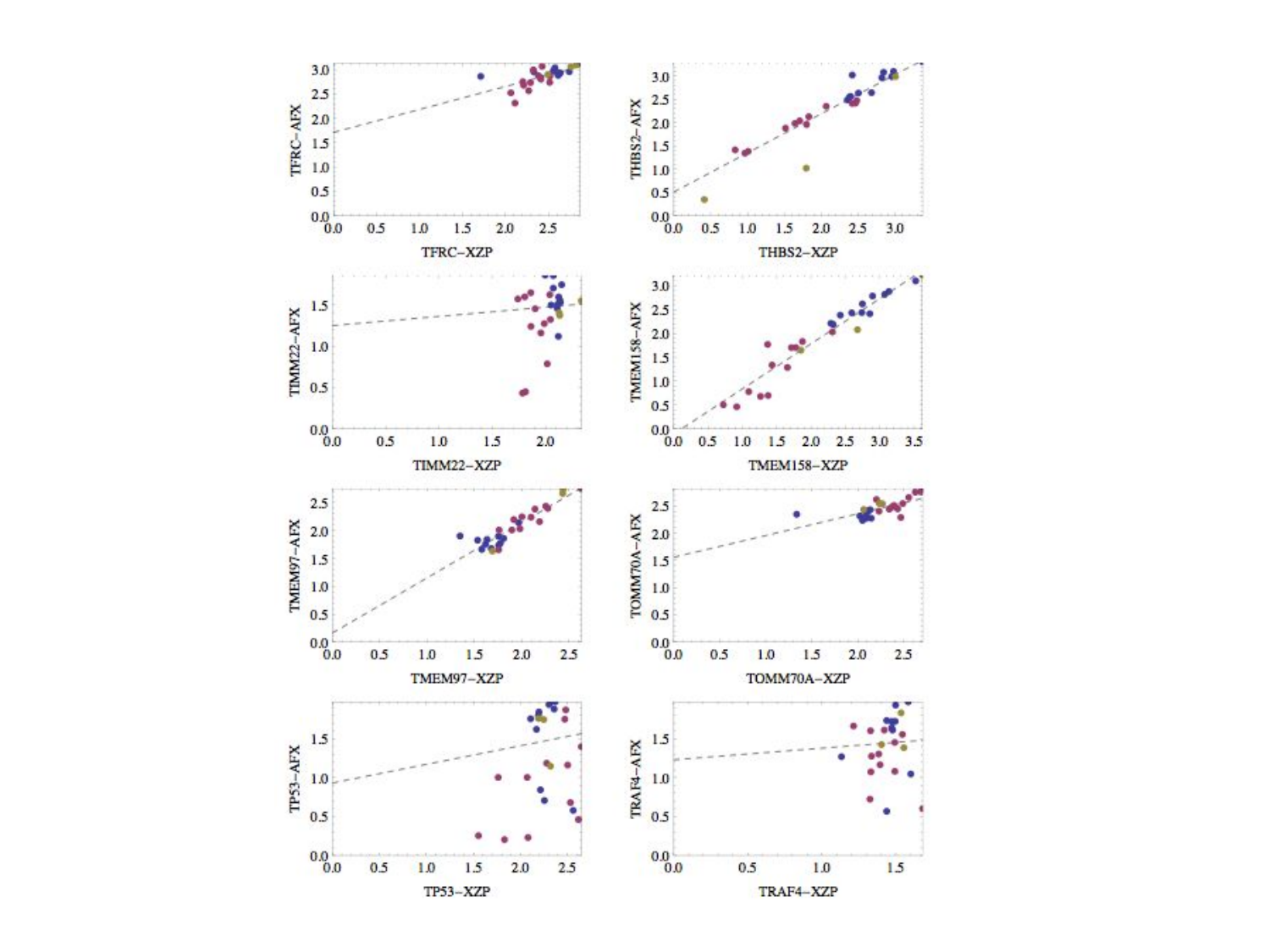

## Slide 12
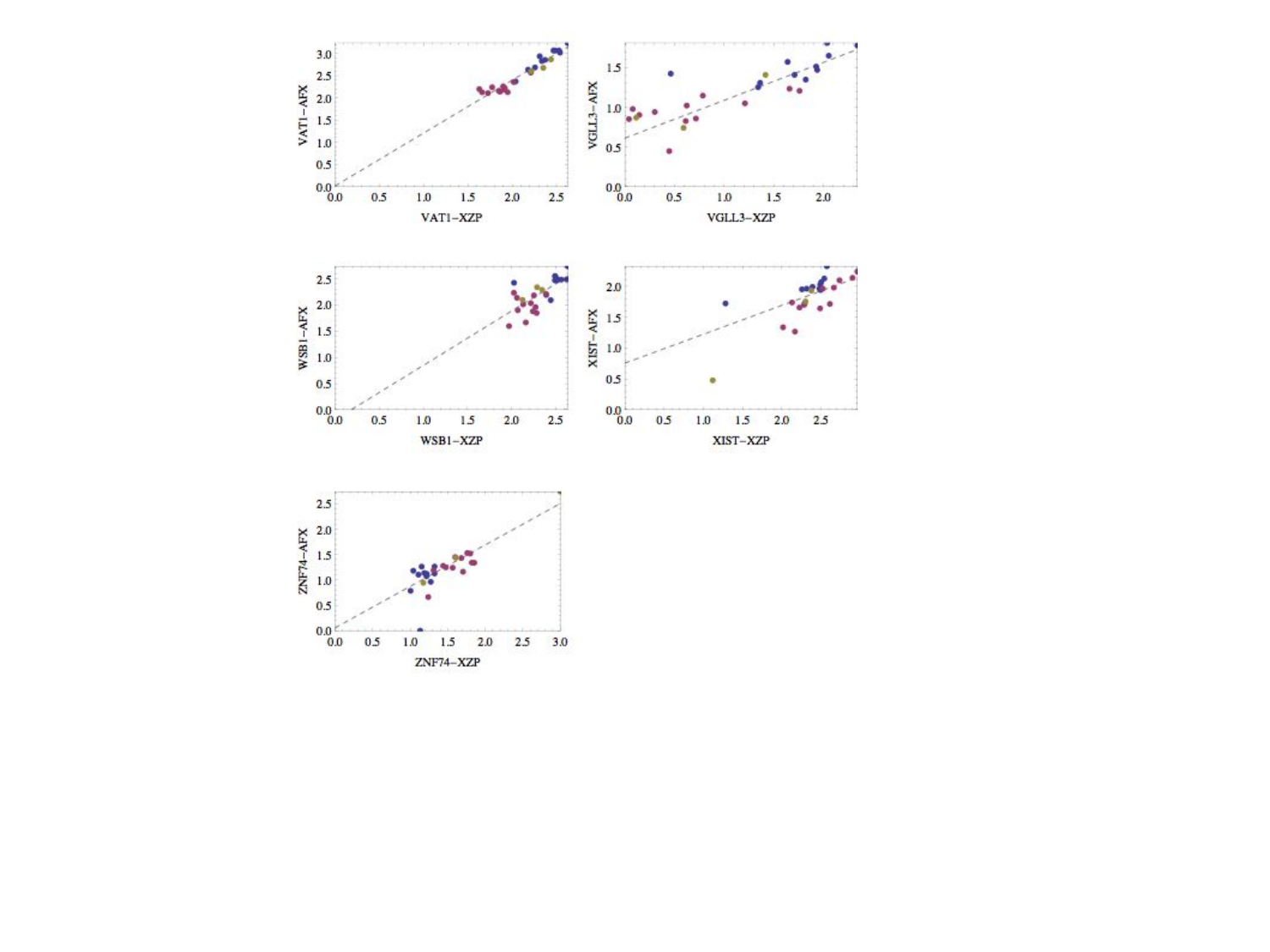

Supplement: Additional file 6 — Correlations between Affymetrix U133A and Xceed Ziplex data. Correlation graphs plotted for all 93 study genes, organized alphabetically. TOV samples are shaded red, NOSE blue and cell lines are indicated in green. [file 1479-5876-7-55-S6.ppt]
